# MuSK agonistic antibody has therapeutic potential for MuSK antibody-positive myasthenia gravis

**DOI:** 10.1016/j.neurot.2026.e01090

**Published:** 2026-10-05

**Authors:** Kentaro Yamamoto, Akiyuki Uzawa, Genta Narazaki, Yoshikazu Nezu, Mami Yamazaki, Hirofumi Kondo, Kozo Yoneda, Masaya Shimada, Chigusa Yoshimura, Yoko Nakano, Manato Yasuda, Hiroyuki Akamine, Yosuke Onishi, Hideo Handa, Etsuko Ogaya, Tomonori Kunikata, Ryutaro Nakashima, Hidenori Takahashi, Tomohiro Nishizawa, Satoshi Kuwabara, Kiyosumi Takaishi

**Affiliations:** a Shinagawa R&D Center, Daiichi Sankyo Co., Ltd., 1-2-58, Hiromachi, Shinagawa-ku, Tokyo, 140-8710, Japan; b Department of Neurology, Graduate School of Medicine, Chiba University, 1-8-1 Inohana, Chuo-ku, Chiba-shi, Chiba, 260-8670, Japan

**Keywords:** Myasthenia gravis, Neuromuscular disease, Autoimmune disease, MuSK signaling

## Abstract

Muscle-specific tyrosine kinase (MuSK)-antibody-positive myasthenia gravis (MG) is caused by MuSK autoantibodies that disrupt acetylcholine receptor clustering at neuromuscular junctions, resulting in muscle weakness and often a severe myasthenic crisis. Given the limited efficacy of conventional treatments involving immunosuppressants and steroids for patients with MuSK-MG, novel treatment approaches have been actively explored. In this study, we investigated the potential of the MuSK agonistic antibody, a novel therapeutic tool, to recover MuSK signaling in the presence of varying levels of autoantibodies. We obtained MuSK agonistic antibodies that bind to the Fz domain of MuSK. The efficacy of the antibody was demonstrated in a MuSK-MG mouse model. The effect of MuSK signaling recovery by our MuSK agonistic antibody was evaluated in the presence of autoantibodies in sera collected from 12 patients with MuSK-MG. We found that a decreasing trend of MuSK signaling recovery by MuSK agonistic antibody was observed upon increasing the serum concentration in some cases. Our findings imply that, for the future clinical development of a MuSK agonistic antibody, there is a need to identify those who will respond well to such an antibody or to develop an add-on treatment to reduce autoantibody levels. This would mitigate the potential risk of insufficient efficacy of such an antibody in patients with MuSK-MG who have high autoantibody concentrations.

## Introduction

Muscle-specific kinase (MuSK)-antibody-positive myasthenia gravis (MG) is a rare autoimmune disease caused by an autoantibody that targets MuSK and blocks neuromuscular signal transmission mainly by disrupting acetylcholine receptor (AChR) clustering. The clinical hallmark of MuSK-MG is fatigable muscular weakness, especially in bulbar muscles, limbs, and the respiratory system, resulting in a higher risk of myasthenic crisis [[1], [2], [3]]. Affected individuals often develop muscle atrophy. Immunosuppressive therapies, including corticosteroids and other immunosuppressive agents, can improve myasthenic symptoms in many patients with MuSK-MG [4,5]. However, achieving minimal manifestation status or better often requires prolonged or high-dose corticosteroid treatment and additional immunosuppressive agents, which can lead to dose-dependent and cumulative adverse effects. Moreover, some patients remain treatment-dependent or refractory [4]. There is thus a need to develop a novel therapy that specifically remedies the pathogenic mechanisms to reduce the potential side effects of prolonged use of steroids or immunosuppressive therapies and achieve lifelong control of severe myasthenic symptoms.

Under physiological conditions, MuSK is activated through tetrameric complex formation with LDL receptor-related protein 4 (LRP4) [6,7]. At neuromuscular junctions (NMJs), agrin, a heparan sulfate proteoglycan secreted by motor neurons, binds to LRP4 and promotes the formation of the agrin/LRP4/MuSK signaling complex. In this complex, LRP4 simultaneously recruits agrin and MuSK, thereby facilitating a direct interaction between agrin and MuSK and activating MuSK signaling [8,9]. This tetrameric complex efficiently induces downstream signaling characterized by the *trans*-phosphorylation of MuSK, docking protein-7 (DOK-7) binding, and clustering of AChRs [10]. In MuSK-MG, pathogenic autoantibodies that mainly recognize the immunoglobulin-like 1 (Ig1) domain of MuSK and interfere with the formation of the tetrameric MuSK complex by inhibiting LRP4–MuSK interaction. This reduces agrin-induced phosphorylation of the intracellular domain of MuSK and impairs downstream signaling required for the formation and maintenance of AChR clusters and NMJ organization, eventually leading to reduced neuromuscular transmission [2,11]. Therefore, impairment of postsynaptic LRP4-MuSK signaling is considered to be the critical pathogenic factor of MuSK-MG. In addition to MuSK-MG, postsynaptic dysfunction of NMJs may be involved in the pathogenesis of spinal muscular atrophy, amyotrophic lateral sclerosis (ALS), autosomal dominant Emery-Dreifuss muscular dystrophy, and some forms of Charcot-Marie-Tooth disease [[12], [13], [14], [15], [16], [17]]. Notably, recent studies have shown that activation of MuSK signaling, either by AAV-mediated DOK-7 gene therapy or by transgene-based overexpression of MuSK protein, increases motor activity and lifespan in the SOD1-G93A ALS mouse [14,17].

Antibody-mediated signal activation is an alternative therapeutic approach for activating the MuSK pathway. It was reported that an anti-MuSK agonistic antibody reversed the muscle defects in mouse models of DOK-7-deficient and agrin-deficient congenital myasthenic syndrome (CMS), as well as in passive-transfer MuSK-MG model [[18], [19], [20], [21], [22], [23]]. Considering the practical application of MuSK-activating antibodies in clinical practice, it is important to understand how the quantities and characteristics of autoantibodies in MuSK-MG patients influence their efficacy. In this context, we generated anti-MuSK agonistic antibodies and evaluated whether they could restore MuSK signaling in the presence of pathogenic autoantibody to MuSK. In particular, we examined their efficacy in a MuSK-MG mouse model and assessed the influence of patient-derived MuSK autoantibodies on agonistic antibody-mediated recovery of MuSK signaling using a human MuSK-expressing myotube model. Our study supports MuSK activation by agonistic antibodies as a promising therapeutic approach for MuSK-MG. It also suggests that level of patient-derived autoantibodies may affect therapeutic efficacy, highlighting the potential importance of patient stratification or combination with standard therapy that reduce autoantibody in patients who respond insufficiently to agonistic antibody therapy.

## Methods

### Study design

This study used animal models and samples from human patients. Age-matched female mice were randomly assigned to groups matched for bodyweight and the titer of antibodies to MuSK. Endpoints were determined on the basis of the established disease model for muscle weakness. Sample sizes for experimental groups were determined on the basis of power calculations using results from our prior investigations in mouse models. The investigator teams were not blinded to the groups of animals. These animal studies were conducted with the approval (No. 20000135) of the Institutional Animal Care and Use Committee of Daiichi Sankyo Co., Ltd. (Tokyo, Japan), and in accordance with this committee's guidelines. Animal *in vivo* studies were performed with 6–8 age-matched female mice per group and *in vitro* studies on 3 to 4 cell cultures from 3 independent experiments. Studies with human samples were performed on 3 to 4 cell cultures per group.

### Sera from patients with MuSK-MG

Twelve Japanese patients with MuSK-MG were included in this study. The diagnosis of MuSK-MG was based on the presence of a clinical history typical of MG with fluctuating muscle weakness accompanied by the presence of serum MuSK antibodies. A non-responder to steroids was defined as a patient with MuSK-MG who experiences recurring deterioration of symptoms despite daily high-dose oral prednisolone treatment or who requires additional immunosuppressive therapy to achieve minimal manifestations. All patients gave written informed consent to participate in this study, and the study was approved by the ethics committees of Chiba University (No. 3624) and Daiichi Sankyo Co., Ltd. (No. 000359–04).

### Active immunization for development of MuSK-MG mouse model

His-tagged hMuSK Ig-1 domain (aa 22–120) was purified by Ni affinity chromatography and size-exclusion chromatography (HILOAD 26/60 SUPERDEX 75 PG; GE Healthcare). Female C57BL/6 mice were obtained from The Jackson Laboratory Japan, Incorporated. Female A/J mice were obtained from CLEA Japan, Incorporated. All mice were used at 7 weeks of age and were housed under specific pathogen-free conditions with a 12-h light/dark cycle and free access to food and water. Animal welfare was assessed by monitoring body weight, general appearance, activity, and signs of distress throughout the study. On day −16, C57BL/6 female mice were anesthetized with isoflurane (Pfizer) and intradermally immunized with 30 μg of the MuSK Ig-1 peptide emulsified with complete Freund's adjuvant (Sigma-Aldrich, #F5881). All mice were subcutaneously boosted on day 0 with 30 μg of MuSK Ig-1 peptide emulsified with incomplete Freund's adjuvant (Sigma-Aldrich, #F5506). On day −13, A/J female mice were anesthetized and intradermally immunized with 6 μg of the MuSK Ig-1 peptide emulsified with complete Freund's adjuvant. All mice were subcutaneously boosted on day 0 with 6 μg of MuSK Ig-1 peptide emulsified with incomplete Freund's adjuvant. Non-immunized mice were injected with an equal volume of PBS in the same manner and schedule. IgG in the sera was measured by ELISA using recombinant rat MuSK-coated plates.

### Antibody development

Rat monoclonal antibodies were generated as previously reported [24]. Briefly, hybridomas producing monoclonal antibodies were generated from rats after immunization with a recombinant hMuSK ECD protein. Antibodies from hybridoma supernatants were selected based on AChR clustering assay and cell ELISA (described in Cell ELISA section). Two selected rat anti-MuSK antibodies were humanized by CDR grafting to minimize immunogenicity [25]. To reduce the effector function of an antibody, a humanized IgG1-formatted antibody clone with heavy-chain mutation (L234 A/L235A) was used for analysis [26]. From this screening, the antibody targeting the Ig1 of MuSK was also identified. This animal study was conducted with the approval (No. A1500220) of the Institutional Animal Care and Use Committee of Daiichi Sankyo Co., Ltd. (Tokyo, Japan), and in accordance with this committee's guidelines.

### Surface plasmon resonance

His-tagged human and mouse MuSK ECD domains were purified by Ni affinity chromatography and size-exclusion chromatography. The binding affinity of Ab#03-H and Ab#40-H against the recombinant MuSK ECD protein was analyzed by SPR using a Biacore T200 system (Cytiva). 0.2 μg/mL purified Ab#03-H or Ab#40-H was injected and immobilized onto a Series S Sensor Chip CM5 (GE Healthcare) using a human antibody capture kit (GE Healthcare). For analysis of Ab#03-H, each point of a 2-fold concentration series of human or mouse MuSK ECD peptide starting at 10 nM was injected. For Ab#40-H, starting concentration of antigens were 20 nM. Dissociation constant (KD) values were calculated in a 1:1 fitting model using Biacore T200 Evaluation software (Cytiva). HBS-EP + buffer (10 mM HEPES, 150 mM NaCl, 3 mM ethylenediaminetetraacetic acid, and 0.05% (v/v) Surfactant P20, pH 7.4) was used as running buffer.

### *In vivo* assessment of neuromuscular function

CMAP was analyzed using a PowerLab/4 S P (ADInstruments). Anesthetized mice were maintained at 37 °C on a thermoregulation pad. Stimulating electrodes were inserted into the muscle near the sciatic nerve for supramaximal stimulation at 40 Hz. Two recording electrodes were inserted into the gastrocnemius muscle or near the Achilles tendon. Finally, a ground electrode was placed in the lumbar region. The decrement was calculated by subtracting the minimum amplitude of CMAPs evoked by a train of 10 pulses from its maximum using LabChart software V7 for Windows (ADInstruments). Wire hanging was measured based on the latency of a mouse to fall off a rod. Mice were allowed to grip the rod with their forelimbs and suspended until they released their grip. The time until falling was recorded over a maximum period of 150 s. Latency time for mice that did not fall from the rod was regarded as 150 s. As soon as the mice fell, the next trial of wire hanging test was conducted. The cumulative latency represents the total time from three consecutive trials.

### Immunostaining and confocal imaging *in vivo*

For quantification of AChR cluster intensity, we performed whole mount staining of the soleus muscle. The soleus muscle was extracted from the right hindlimb and fixed in 1% Paraformaldehyde (PFA)/PBS for 2-h at room temperature. It was then washed with PBS and incubated with 0.1 M glycine in PBS for 30 min, followed by incubation with blocking solution (2% bovine serum albumin and 1% Triton-X in PBS) at 4 °C overnight. Muscle samples were then exposed to a staining mixture of Alexa Fluor 594-conjugated α-bungarotoxin (Invitrogen, #B13423) to label AChRs and Alexa Fluor 488-conjugated anti-synapsin-1 antibody (Cell Signaling Technology, #13197) overnight at 4 °C. Each muscle was then washed with PBS and flattened on a slide glass. Finally, the samples were mounted with VectaShield (Vector Laboratories, #H-1400-10) for confocal microscopic analysis. Confocal images were acquired using a YOKOGAWA CQ1 image cytometer and quantified on a maximum-intensity projection of the images using CQ1 software (YOKOGAWA).

### *In vitro* AChR clustering assay

C2C12 and HSMM cells, purchased from ATCC (CRL-1772) and Lonza (CC-2580), respectively, were maintained at 37 °C in Dulbecco's modified Eagle's medium (DMEM) supplemented with 20% fetal bovine serum (Gibco). Myotube differentiation was induced by switching to DMEM with 2% horse serum (Gibco) when cells reached confluence on 96-well glass plates (Grainer, #655981). Six days after differentiation, 0.7, 7, and 70 μg/mL Ab#40-H with or without 10 ng/mL agrin (R&D Systems, #550AG) were administered for 8-h to induce AChR clustering, followed by 1 h of incubation with α-bungarotoxin-Alexa 488 (Invitrogen, #B13422). The samples were fixed in formalin for 15 min at room temperature. For quantification of AChR clustering, nine predefined fields of view were acquired from each well and defined as ROIs. These fields approximately covered the entire well area. AChR cluster signals within the nine ROIs were quantified using identical acquisition and analysis settings, and the total signal from the nine fields was used for analysis. All samples were analyzed using the IN Cell Analyzer 6000 and IN Cell Investigator software (Cytiva).

### MuSK tyrosine phosphorylation assay

HSMM cells were stimulated with 100 ng/mL Rat agrin (R&D Systems, #550AG) or 0.01, 0.1, or 1 μg/mL Ab#40-H for 1 h. For the precipitation of MuSK, the lysate was mixed with the complex of Protein G Sepharose and anti-MuSK capture antibody (in-house) at 4 °C overnight. The bead-precipitated proteins were detected by immunoblotting after incubation at 95 °C for 10 min with sample buffer. Anti-MuSK antibody (R&D Systems, #AF562, 1:1000) and anti-pMuSK antibody (SAB4301394, Sigma 1:200) were used as primary antibodies to detect MuSK protein and phosphorylated MuSK, respectively.

### Measurement of serum anti-MuSK IgG titers by ELISA

Serum anti-MuSK IgG titers were measured by ELISA using recombinant rat MuSK-coated plates. Briefly, 96-well plates were coated with recombinant rat MuSK protein (in-house; 0.1 μg/mL in PBS, 200 μL/well) overnight at 4 °C. Plates were washed with PBST (PBS containing 0.05% Tween-20) and blocked with PBST containing 1% bovine serum albumin for 1 h at room temperature. Mouse serum samples diluted 1:5000 in blocking buffer were added at 100 μL/well and incubated for 1 h at room temperature. After washing, bound mouse IgG was detected with HRP-conjugated anti-mouse IgG antibody (1:6000; GE Healthcare, #NA931V-1 ML) for 1 h at room temperature. TMB soluble reagent was then added at 100 μL/well and incubated for 30 min at room temperature. The reaction was stopped with TMB stop buffer, and absorbance at 450 nm was measured using a SpectraMax M5e microplate reader (Molecular Devices).

### Cell ELISA

293 A cells were purchased from and authenticated by Thermo Fisher Scientific (R70507). Cells were grown at 37 °C in the same medium as described for C2C12 myoblasts. Full-length hMuSK (NM_005592) was cloned into pFLAG-19 (Sigma-Aldrich) vector (Fw primer: 5′-ttgcggccgcgaattcaaaagctcctgtcatcac-3′, and Rv primer: 5′-atctatcgatgaattgacactcacagttccctc-3′). Truncation mutants of the Ig-1 domain (Fw: 5′-ttgcggccgcgaattccctgcaagtgaagatg-3′), of Ig-1 and Ig-2 domains (Fw: 5′-ttgcggccgcgaatttgaagctggaagttgagg-3′), and of Ig-1, Ig-2, and Ig3 domains (Fw: 5′-ttgcggccgcgaattccatagcagaatggagtaaacc-3′) were subcloned into pFLAG-19 using an In-Fusion HD Cloning Kit (Takara). These plasmids were transfected into 293 A cells in collagen-coated 96-well plates (Sumitomo Bakelite) using Lipofectamine 2000 Transfection Reagent (Life Technologies). The antibodies or sera were diluted in PBS containing 2% fetal bovine serum and applied to the 293 A cells expressing the hMuSK and hMuSK truncation mutants for 1 h at 4 °C without fixing the cells. The supernatant was washed three times with cold PBS and treated with anti-human IgG antibody conjugated with HRP (Proteintech, #SA00001-17, 1:1000) for 1 h at 4 °C. After three washes, 100 μL of TMB substrate solution (ScyTek Laboratories, #TM4500) was added to each well for 4 min, after which 100 μL of TMB stopping buffer (ScyTek Laboratories, #TSB500) was added to stop the enzyme reaction. The absorbance at 450 nm was measured using a SpectraMax M4 plate reader (Molecular Devices).

### Differentiation of C2C12 myotube expressing hMuSK and AChR assay

The mMuSK-KO C2C12 cell line was generated using a complex of Cas9 and a single guide RNA targeting exon 2 on chromosome 4 (Fw: 5′-ctcttaaatacagacccagaagtgg-3′, Rv: 5′-gcccatgtgcaataagagcaatcag-3′). The complex was co-transfected by electroporation (Lonza, Nucleofector). Candidate single clones were verified by genomic sequencing, and we confirmed that the selected C2C12-mMuSK KO had a 27-bp deletion in intron 1 on one allele and a 340-bp deletion of intron 1 and intron 2 including the entire sequence of exon 2 on the other allele. Immunoblot analysis also confirmed that protein bands of neither the full-length MuSK protein nor the fragmented product of MuSK were detected (R&D Systems, #AF562, 1:1000). mMuSK-KO C2C12 cells were maintained and differentiated in the same medium as described for C2C12 myoblasts. Two days after differentiation, 4 nM IGF-1 (PeproTech, #100–11) was added, and 4 days after differentiation, mRNA encoding hMuSK (in-house) was transfected into the mMuSK-KO myotube (Invitrogen, # LMRNA015). On day 5, an *in vitro* AChR clustering assay was performed.

### Competitive binding assay

Ab#40-H was labeled with biotin using the Biotin Labeling Kit, NH2 (DOJINDO, #LK03), in accordance with the manufacturer's instructions. Sera were diluted serially in PBS containing 2% fetal bovine serum (Gibco) and applied to 293 A cells expressing hMuSK for 1 h at 4 °C without fixing the cells with 7 μg/mL Ab#40-H. The supernatants were washed three times with 300 μL of cold PBS, followed by the application of streptavidin-HRP (Abcam, #ab7403) for 15 min. After washing three times with 300 μL of cold PBS, 100 μL of TMB substrate solution (ScyTek Laboratories, #TM4500) was added to each well for 5 min. Subsequently, 100 μL of TMB stopping buffer (ScyTek Laboratories, #TSB500) was added to stop the enzyme reaction. Absorbance was measured at 450 nm using a SpectraMax plate reader (Molecular Devices).

### Statistical analysis

Statistical analysis was performed in REDPOST/BI (SAS System Release 9.2; SAS Institute Inc., Cary, NC, USA). For *in vitro* assays, the significance of differences was determined using parametric Dunnett's test, Student's t-test, Welch's *t*-test, and Shirley-Williams test. A two-sided P-value of <0.05 was considered statistically significant.

## Results

### Development of a novel anti-MuSK agonistic antibody

We immunized rats with recombinant human MuSK (hMuSK) protein and screened for antibodies that activate MuSK downstream signaling from hybridoma supernatants. Specifically, antibodies were selected by their ability to bind to hMuSK and induce AChR clustering in C2C12 myotubes in a manner independent of agrin (Fig. 1A). For future therapeutic use in humans, further selection was performed based on the compatibility of their CDR sequences with human acceptor frameworks for CDR grafting [25,26]. Ultimately, we selected two lead clones (Ab#03-R and Ab#40-R) and their humanized counterparts were designated Ab#03-H and Ab#40-H, respectively. As part of the screening and selection process, we further evaluated the agonistic activities of the humanized antibodies and their corresponding original antibodies using human skeletal muscle myoblast (HSMM) myotubes. Ab#03-H increased the number of AChR clusters in HSMM myotubes to a level comparable to that induced by its originator antibody, while Ab#40-H showed higher activity to induce AChR clustering than its original antibody under the same condition (Fig. 1B). Furthermore, Ab#03-H and Ab#40-H increased MuSK phosphorylation in a dose-dependent manner (Fig. 1C). Finally, monovalent binding of either Ab#03-H or Ab#40-H was determined by surface plasmon resonance (SPR). Ab#03-H recognized both hMuSK and mouse MuSK (mMuSK) with high picomolar affinities (0.22 and 0.78 nM, respectively) and showed strong cross-reactivity between hMuSK and mMuSK (Fig. 1D). In addition, Ab#40-H showed slightly lower affinities for hMuSK and mMuSK (13.8 and 16.9 nM, respectively) than Ab#03-H did (Fig. 1D). These results showed that we successfully developed two novel humanized anti-MuSK agonistic antibodies that induced human and mouse MuSK signaling in an agrin-independent manner.

**Fig. 1:**
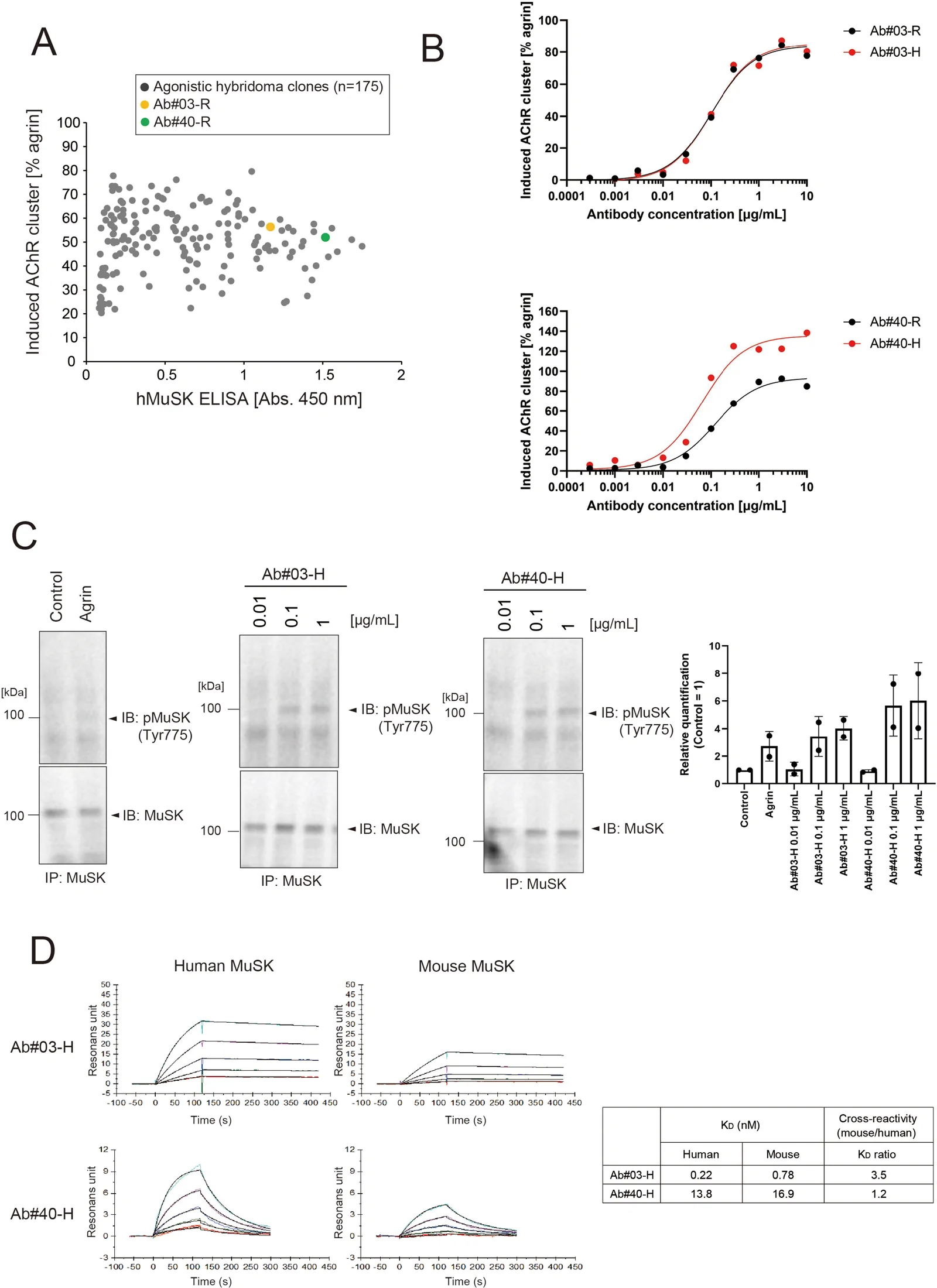
**A novel anti-MuSK agonistic antibody was selected by affinity and agonistic activities to hMuSK.** (**A**) Scatter plot analysis of affinity to hMuSK and potential for AChR clustering of each hybridoma supernatant. Binding to hMuSK was measured by ELISA. The potential for AChR clustering was evaluated by the AChR clustering assay in C2C12 myotube. The y-axis shows the number of AChR clusters in ROI, normalized to the response induced by 10 ng/mL agrin. (**B**) Humanized and original antibodies of Ab#03 and Ab#40 were compared in the AChR clustering assay. The y-axis showed the number of AChR clusters in ROI normalized to the response induced by 10 ng/mL agrin. (**C**) HSMM cells were treated with agrin, Ab#03-H or Ab#40-H and MuSK phosphorylation at Tyr775 was analyzed by immunoblotting after immunoprecipitation of each lysate with MuSK antibody. The Right graphs, relative expression levels of phosphorylated MuSK, with the control set to 1. The bar graphs show the mean ± S.D. (n = 2) (**D**) SPR sensorgram of Ab#03-H and Ab#40-H binding to recombinant human or mouse MuSK. Here, 1.25 nM–20 nM solutions of the extracellular domain of MuSK were prepared by serial dilution at a ratio of 1:2 and injected. K_D_ value was determined by 1:1 fitting models and cross-reactivity between mouse and human MuSK was calculated by dividing human K_D_ by mouse K_D_.

### Ab#40-H fully activates MuSK signaling

MuSK-ECD carries three extracellular immunoglobulin (Ig)-like domains (Ig1, Ig2, and Ig3) and one frizzled (Fz)-like domain. To determine which domain Ab#40-H recognizes, we performed cell enzyme-linked immunosorbent assay (ELISA), which evaluated the binding affinity of the antibody to 293 A cells expressing full-length MuSK or MuSK lacking Ig1, Ig1 and Ig2, or Ig1, Ig2, and Ig3 (ΔIg1, ΔIg1-2, and ΔIg1-3, respectively). 293 A cells showed little or no endogenous MuSK expression, and comparable expression levels of full-length and truncated hMuSK constructs were confirmed by immune blotting (Supplementary Fig. 1A). Ab#40-H exhibited binding to all of the cells expressing these MuSK-ECD truncation mutants, suggesting that it binds to the Fz domain of MuSK (Fig. 2A). In the process of selecting MuSK agonistic antibodies from MuSK-immunized rats, we identified some antibodies that inhibit the agrin-induced MuSK signaling (Supplementary Fig. 1B). We characterized one of these antibodies by cell ELISA and demonstrated that it bound only to cells expressing full-length MuSK (Fig. 2A), suggesting that it binds to the MuSK Ig1 domain. Given that most autoantibodies in MuSK-MG patients bind to the Ig1 domain, whether a MuSK antibody exerts an agonistic effect on MuSK signaling or an antagonistic one was considered to be largely dependent on the target domain, and binding to the Ig1 domain and to the Fz domain are characteristic of MuSK antagonistic and agonistic antibodies, respectively [11,21,27].

**Fig. 2:**
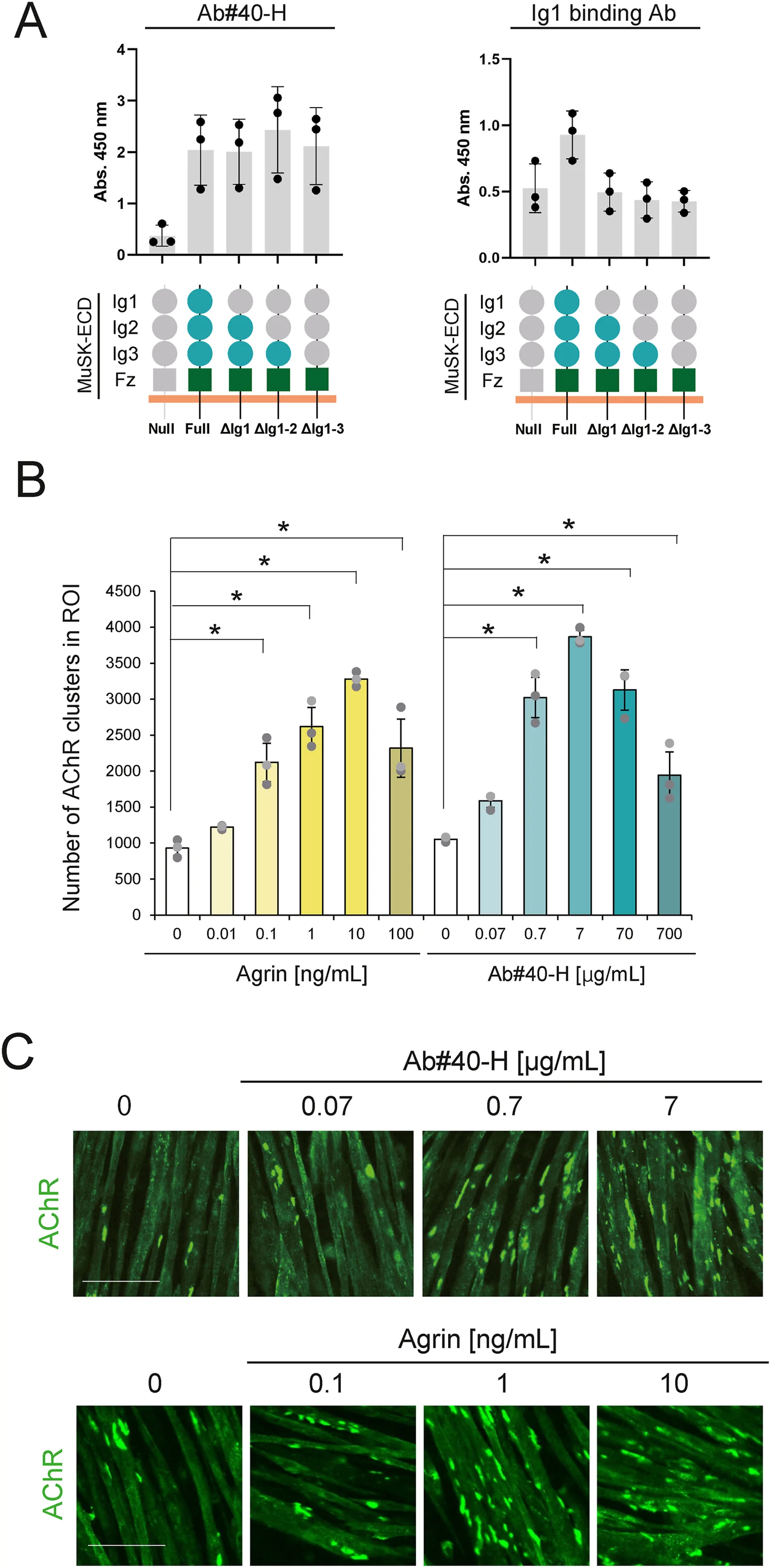
**MuSK agonistic antibody increased AChR clustering by binding to the Fz domain.** (**A**) Binding of Ab#40-H (left) or the anti-MuSK antagonistic antibody (right) to 293 A cells expressing full-length or truncated MuSK protein was analyzed by cell ELISA for domain mapping. Schematic illustration of the full-length and truncated MuSK is shown. (**B**) Agrin and Ab#40-H induced AChR cluster formation in C2C12 myotubes. Agrin at 10 ng/mL (47.9 pM) induced AChR clustering to levels comparable to those induced by 0.7 μg/mL (4.67 nM) of Ab#40-H. (**C**) AChR was stained with fluorophore-conjugated α-bungarotoxin (green) and the number of AChR clusters in the ROI was counted. Representative confocal images of AChR are shown (bottom). Scale bar, 200 μm. All bar graphs present mean ± S.D. (n = 3, Dunnett's test between untreated and Ab#40-H-treated groups; ∗P < 0.05).

Next, to assess the potential of Ab#40-H to induce AChR clustering compared with that of agrin, an AChR clustering assay was performed using C2C12 myotubes. AChR clustering on C2C12 myotubes showed a bell-shaped dose-response curve to agrin (Fig. 2B and Supplementary Fig. 1C). Interestingly, AChR clustering on C2C12 myotubes also showed a bell-shaped dose-response curve to Ab#40-H (Fig. 2B and Supplementary Fig. 1C). In addition, our immunocytochemistry data illustrated that Ab#40-H treatments increased the formation of AChR clustering in C2C12 myotubes independent of agrin treatments (Fig. 2C). At the concentration inducing the highest response, Ab#40-H induced almost the same level of AChR clustering as agrin, suggesting strong potential of Ab#40-H as an agonist of MuSK signaling.

### The pharmacokinetic properties support twice-weekly administration for *in vivo* studies

We next investigated the therapeutic potential of our anti-MuSK agonistic antibodies in a MuSK-MG disease model. To determine the dosing regimen, we subcutaneously administered the MuSK agonistic antibody at 10 mg/kg to A/J mice and analyzed the pharmacokinetic (PK) properties. The half-life of Ab#03-H and Ab#40-H in circulation was around 10 days and their maximum plasma concentrations reached 100.4 and 72.8 μg/mL, respectively (Supplementary Fig. 2). Since the PK profile showed no evidence of nonlinear clearance due to target-mediated drug disposition, the concentration of both MuSK agonistic antibodies remained above 10 μg/mL at 15 days post-injection. Based on the pharmacokinetic properties and the higher affinity of Ab#03-H for mouse MuSK, as indicated by its lower KD value compared with Ab#40-H, we decided to administer of Ab#03-H twice a week for *in vivo* study.

### MuSK signal stimulation recovers muscle weakness in MuSK-MG mice

Previous studies reported that the MuSK-MG mouse model established by immunization with the ectodomain of MuSK or full-length MuSK protein showed both muscle weakness and body weight loss, likely reflecting a severe phenotype associated with higher autoantibody levels [28,29]. While body weight loss can also occur in MuSK-MG patients with bulbar symptoms, such as dysphagia and masticatory weakness, acute body weight loss in mice affected the general condition of animals and compromised the reproducibility of wire-hanging test results in our pilot experiments. Therefore, to generate a milder and more stable disease model suitable for evaluating the therapeutic effects of MuSK agonistic antibodies, we modified the immunization protocol by using a synthetic Ig1 domain peptide (amino acids 22–120) of hMuSK, which is a major domain recognized by autoantibodies in MuSK-MG patients, instead of using full-length MuSK protein or larger recombinant MuSK fragments (Fig. 3A) [11]. The production of anti-MuSK IgG antibodies was confirmed in all mice by ELISA within 5 days after booster immunization, and the level of the autoantibodies increased over time thereafter, while no anti-MuSK IgG antibody was detected during the study period in non-immunized mice injected with phosphate-buffered saline (PBS) instead of the peptide (Fig. 3B). Isotype analysis of sera from the immunized mice showed an increase in total serum IgG2a levels after Ig1 peptide immunization (Supplementary Fig. 3). After booster immunization, muscle strength of the mice was evaluated every week for 5 weeks by a wire hanging test. A decrease in muscle strength compared with that of non-immunized mice was observed in all Ig1-immunized mice (n = 8) on day 6 and thereafter (Fig. 3C and Supplementary Fig. 4A). Interestingly, no significant difference in body weight was observed between the model mice and non-immunized mice during the study period (Fig. 3C). The administration of Ab#03-H at 10 and 30 mg/kg significantly inhibited the progressive muscle weakness and muscle function was maintained until the end of the study (33 days after booster immunization) (Fig. 3D). Furthermore, we analyzed the gastrocnemius muscle of the hindlimbs electromyographically. While no significant reduction in compound motor action potential (CMAP) was observed upon 10 repetitive stimulations at 40 Hz in non-immunized mice, the same stimulation decreased CMAP of the muscle in the Ig1 domain peptide-induced MuSK-MG model mice, suggesting that anti-MuSK autoantibodies bind to MuSK at NMJs and block neuromuscular signaling (Fig. 3E and F). Although not statistically significant, administration of Ab#03-H at 10 and 30 mg/kg twice a week suppressed the decrement of CMAP (Fig. 3E and F). Taking these findings together, our anti-MuSK agonistic antibody showed therapeutic potential to prevent the progression of muscle weakness in the MuSK-MG mouse model by restoring MuSK signaling suppressed by anti-MuSK autoantibodies.

**Fig. 3:**
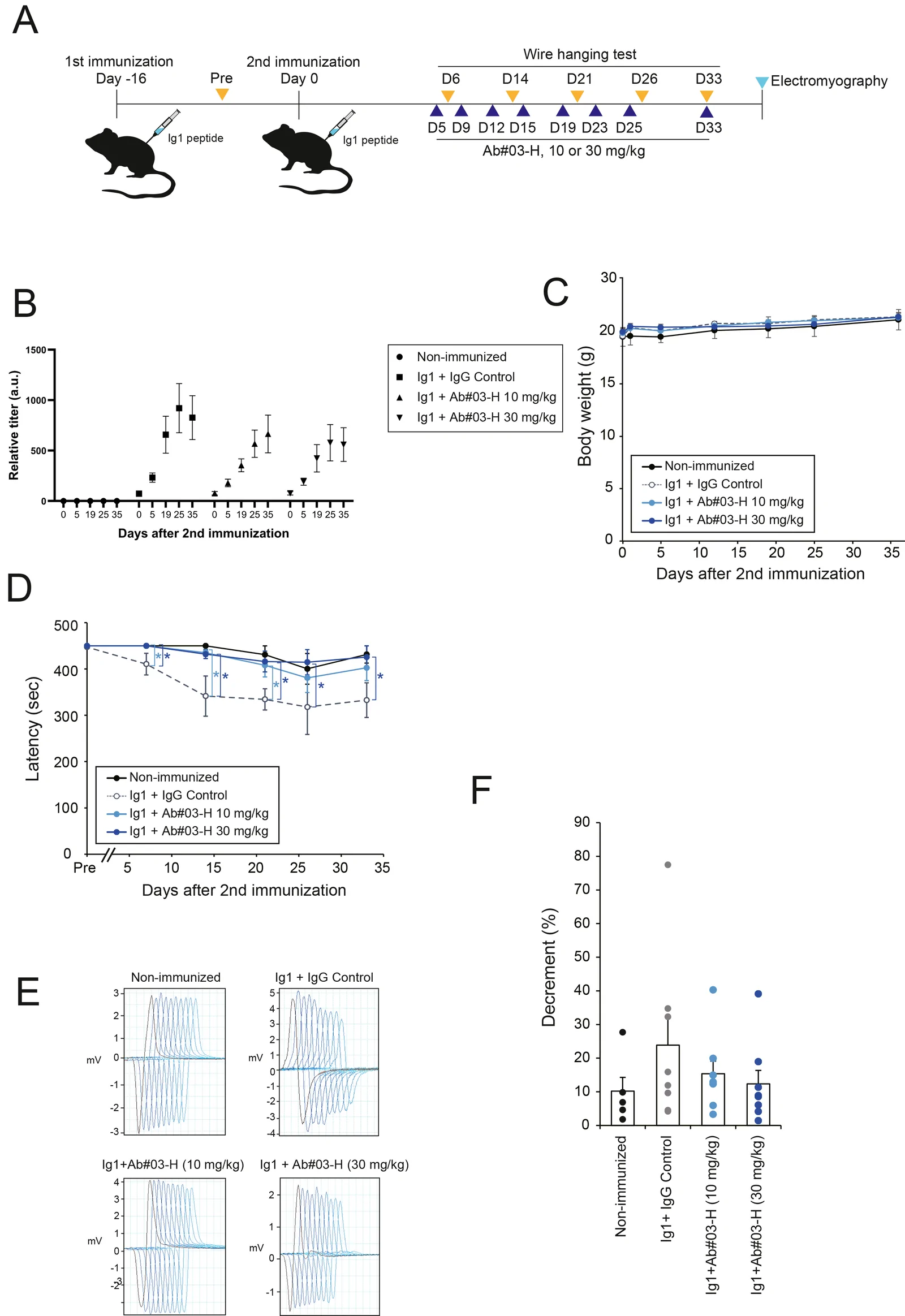
**Ab#03-H sustained the neuromuscular symptoms in MuSK-MG model mice.**(**A**) Experimental design to analyze the effects of Ab#03-H in MuSK-MG model mice. C57BL/6 mice immunized with Ig1 peptide were treated with Ab#03-H or IgG control by twice-weekly *s.c.* injection. Muscle strength was analyzed by wire hanging test and electromyography. (**B**) Anti-MuSK antibody in the serum of mice immunized with the MuSK Ig1 peptide or non-immunized mice was measured by ELISA. The graph presents mean ± S.E. (n = 8 in the MuSK-immunized group and n = 5 in non-immunized group). (**C**) body weight of MuSK-immunized and non-immunized mice was measured over time after the second immunization. The graphs show mean ± S.E. (n = 8 in each of the immunized groups and n = 5 in non-immunized group, Shirley-Williams test between Ig1+IgG control-treated and Ig1+Ab#03-H-treated groups; ∗P < 0.05). (**D**) Muscle strength was evaluated by wire hanging test over time after the second immunization. (Shirley-Williams test between Ig1+IgG control-treated group and Ig1+Ab#03-H-treated groups; ∗P < 0.05). (**E**) Amplitude of 10th CMAP was recorded in the hindlimb during 40 Hz nerve stimulation at the indicated timepoints, and (**F**) the signal decrement was calculated. The graphs display the mean ± S.E. (n = 8 in each of the immunized groups and n = 5 in the non-immunized group).

### MuSK activation maintains NMJ structures

To examine whether the administration of the anti-MuSK agonistic antibody to MuSK-MG model mice can prevent the collapse of the NMJ structure caused by autoantibodies, we performed a series of immunohistochemical staining studies using soleus muscle. In these studies, A/J mice, which have been reported to show a higher incidence of NMJ disruption upon immunization with full-length MuSK protein [28], were immunized with the hMuSK Ig-1 peptide to create a MuSK-MG model (Fig. 4A). In A/J mice, wire-hanging latency was decreased on day 4 and a reduction in CMAP amplitude was observed on day 7 without body weight loss (Supplementary Fig. 4, B-E). Treatment with Ab#03-H significantly improved the reduction of in CMAP amplitude (Supplementary Fig. 4F). However, no difference in the mean intensity of AChR signals at NMJ in soleus muscle was observed between naïve and immunized mice on day 7 (Supplementary Fig. 4G). Therefore, we performed NMJ structural analysis on day 12 using the same dosing regimen as that used in the C57BL/6 mice model.

**Fig. 4:**
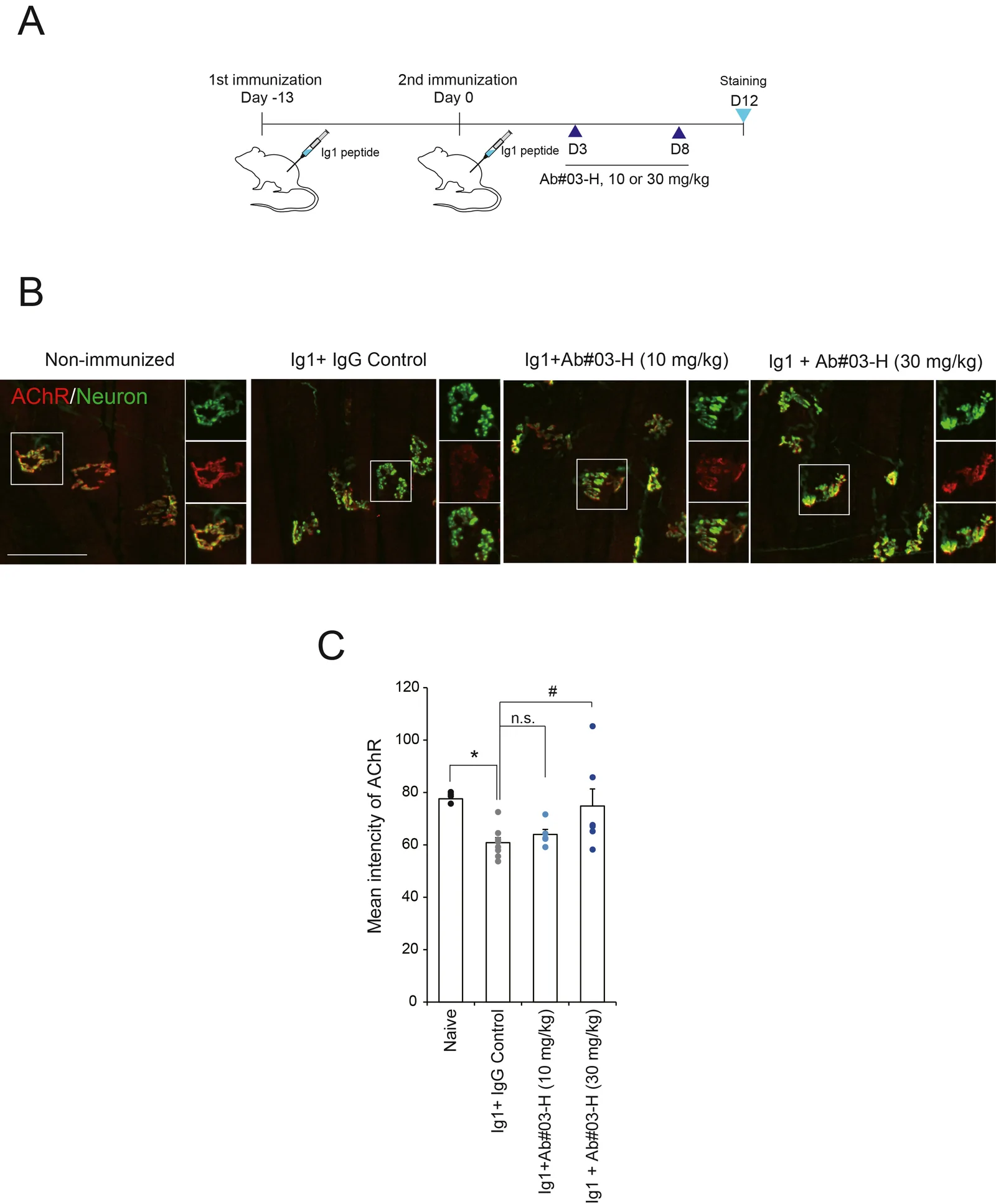
**Anti-MuSK agonistic antibody recovered the NMJ structures in MuSK-MG model mice.**(**A**) Experimental design to analyze the effects of Ab#03-H in MuSK-MG model mice. A/J mice immunized with Ig1 peptide were treated with Ab#03-H or IgG control by twice-weekly *s.c.* injection. NMJ structure in the soleus muscle was analyzed by immunohistochemical staining. (**B**) Representative confocal images of isolated soleus muscle from non-immunized and the MuSK-immunized mice. AChRs (red) and neurons (green) were stained with α-bungarotoxin and anti-synapsin-1 antibody, respectively. Scale bar, 100 μm. (**C**) Quantification of the AChR accumulation in isolated whole soleus muscle in each group. The bar graph presents mean ± S.E. (n = 5–8 in each group, Shirley-Williams test between vehicle- and Ab#03-H-treated groups: #P < 0.05 and n.s., no significance; Welch's *t*-test between non-immunized group and Ig1+IgG control-treated group: ∗P < 0.05).

Soleus muscle from immunized A/J mice was stained with a mixture of α-bungarotoxin and anti-synapsin-1 antibody to analyze the NMJ structure. To minimize variations in the obtained results resulting from the particular view analyzed, Z-series images were captured with a confocal microscope and collapsed into single maximum-intensity projection images. In addition, all NMJs in a whole soleus muscle section were analyzed for intensity calculations. In the mice injected with PBS, NMJs exhibited typical pretzel morphology and AChRs were completely colocalized with neurons (Fig. 4B). However, in the MuSK-MG disease model, punctate structures of nerve terminals were observed, and the intensity of AChR clusters at NMJs was significantly reduced (Fig. 4B and C). Meanwhile, the mean intensity of AChR clusters in soleus muscle from MuSK-MG mice receiving 30 mg/kg Ab#03-H twice weekly was significantly higher than that of MuSK-MG mice receiving the control IgG (Fig. 4C). Interestingly, the mean intensity of AChR clustering in soleus muscle of MuSK-MG mice receiving 30 mg/kg Ab#03-H was nearly identical to that of non-immunized mice (Fig. 4B and C). These findings provide evidence that the prevention of muscle weakness progression by the MuSK agonistic antibody resulted from maintenance of the NMJ structure.

### Ab#40-H enhances AChR clustering in the presence of 1% MuSK-MG sera

To further explore the therapeutic potential of our antibodies for MuSK-MG patients whose autoantibodies block MuSK signaling, we aimed to evaluate the binding of our agonistic antibody to MuSK and AChR clustering in the presence of either sera or purified autoantibodies from MuSK-MG patients. First, we collected 12 serum samples from MuSK-MG patients for whom clinical information, including sex, age, disease duration, MuSK antibody level, immune treatment, steroid response, and disease severity (MGFA class at the time of blood sampling), was available (Table 1). Notably, all patients, apart from No. 3 and No. 11, underwent several kinds of immune treatments to control the myasthenic symptoms, and patients No. 1, 2, 5–7, 9, and 10 were classified as non-responders to steroids. As shown in Fig. 5A, the cell ELISA with 293 A cells expressing full-length and truncated MuSK ECDs indicated that the serum from each patient contained antibodies recognizing different epitopes: eight recognized the Ig1 domain, while two (No. 6 and No. 11) recognized both Ig1 and Ig2. These results were in line with a previous report showing that MuSK autoantibodies predominantly recognize the Ig1 domain of MuSK [11,30]. Total antibody concentrations in two serum samples from patients No. 4 and No. 10 were below the detection limit of a standard clinical laboratory test, and no immunoreactivity was observed in the cell ELISA, either.

**Table 1:** Clinical characteristics of 12 patients with MuSK-MG.

Patients No.	1	2	3	4	5	6	7	8	9	10	11	12
Disease duration, y	28	33	1.1	9	17.4	10.7	6.6	11.1	12.2	27.5	0.7	4.4
MuSK antibody, nmol/mL	21	70	73	4.1	16.8	36.9	14.6	6.85	54	0.02	92	7.55
Immunosuppressants	PSL, TCR,CyA	PSL,TCR,CyA, IVIG, DFPP	PSL	IVIg, DFPP	PSL,TCR,CyA, AZA,IVIg, DFPP	PSL,TCR	PSL,TCR	PSL,DFPP	PSL,TCR,IVIg, PP,AZA,RTX	PSL,TCR,PP		PSL
Steroids response	N	N	Y	Y	N	N	N	Y	N	N	Y	Y
MGFA classification	4b	5	3a	2a	4a	3b	3b	1	3a	3a	2b	0

PSL, Prednisolone TCR, Tacrolimus CyA, Cyclosporine A IVIg, Intravenous immunoglobulin DFPP, Double Filtration Plasmapheresis PP, Plasmapheresis AZA, Azathioprine RTX, Rituximab.

**Fig. 5:**
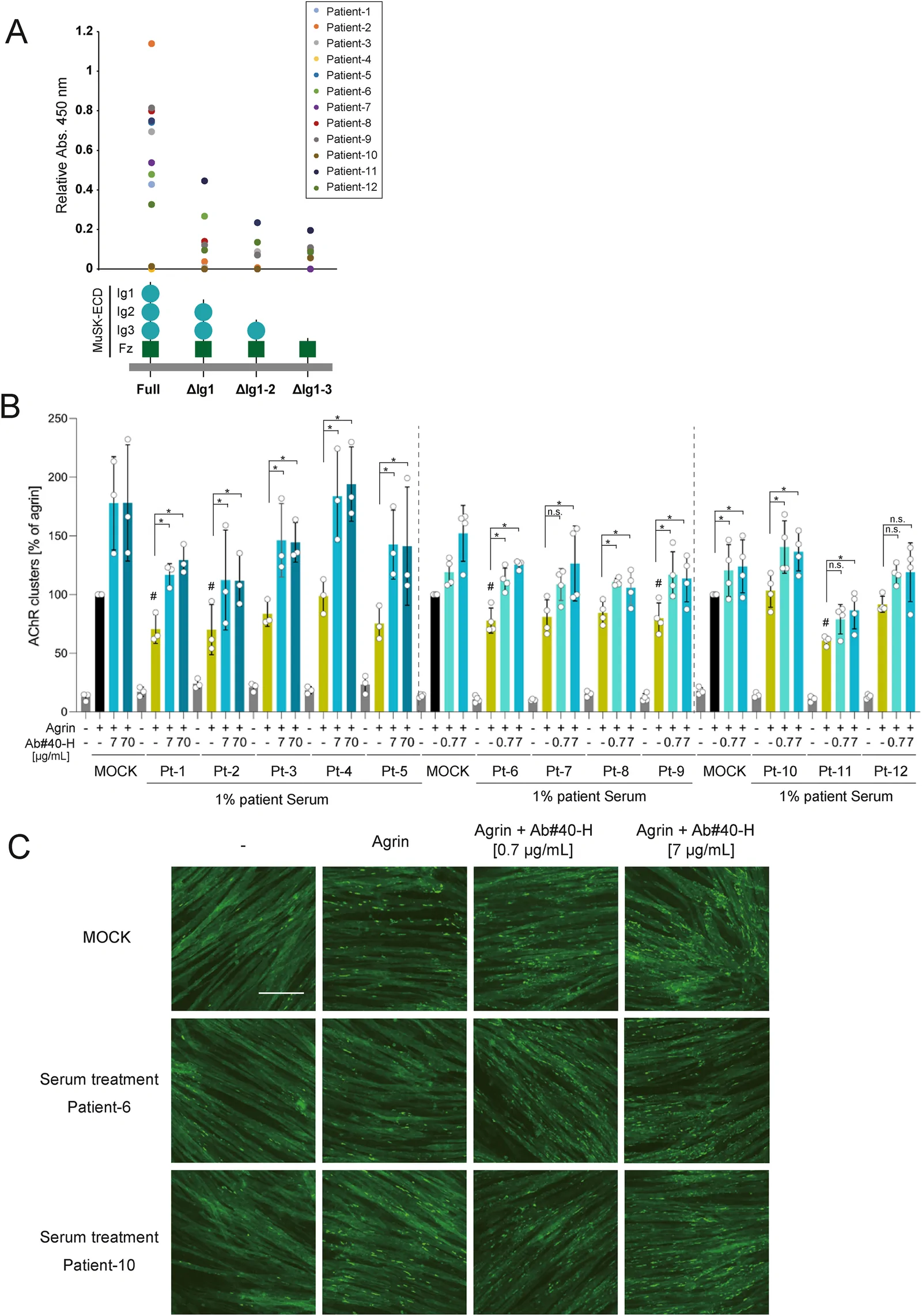
**Anti-MuSK agonistic antibody significantly recovered the AChR clustering in the presence of autoantibody from MuSK-MG patients.**(A) Binding of autoantibodies in 12 serum samples from patients with MuSK-MG to 293 A cells expressing full-length or truncated MuSK proteins was analyzed by cell ELISA for domain mapping. Schematic illustration of the full-length and truncated MuSK is shown. (B) 9 image fields per well (96-well format) were acquired under each condition, and the number of AChR clusters was counted and summed. Among sera from 12 MuSK-MG patients, 5 serum samples significantly inhibited agrin-induced AChR clustering in the C2C12 myotube expressing hMuSK (Dunnett's test between agrin-treated group and agrin and serum-treated group; #P < 0.05), while the addition of Ab#40-H induced AChR cluster formation irrespective of the presence of the serum, except for in Patient No. 12. The bar graphs show the mean ± S.D. (n = 3 or 4, Dunnett's test between serum- and Ab#40-H-treated groups; ∗P < 0.05 and n.s., no significance). (**C**) Representative images of AChR clusters on humanized C2C12 myotubes. In the presence of serum from patient-6, AChR cluster formation induced by agrin was significantly suppressed, whereas serum from patient-10 had little effect. AChR cluster was stained with fluorophore-conjugated α-bungarotoxin (green). Scale bar, 200 μm. Other images were shown in Supplemental Fig. 6.

Next, to evaluate the potential of Ab#40-H antibody to induce MuSK signaling in the presence of hMuSK-targeting autoantibodies in sera from MuSK-MG patents, AChR clustering assay was performed using C2C12 myotubes expressing hMuSK. For this assay, we modified the conventional C2C12 myotube assay by transiently expressing hMuSK in mMuSK-knockout (KO) C2C12 cells generated by CRISPR/Cas9. This modification enables us to evaluate both the inhibitory activity of patient-derived autoantibodies and the agonistic activity of Ab#40-H on AChR clustering, while accounting for potential species differences in antibody–antigen interactions. The expression of hMuSK was confirmed by immunoblotting, and the ability of hMuSK expression to restore AChR clustering in mMuSK-KO C2C12 myotubes was confirmed by immunofluorescence analysis (Supplementary Fig. 5, A-D). While agrin-dependent AChR clustering disappeared in the mMuSK-knockout C2C12 cells, AChR clustering was observed by transient expression of hMuSK in these cells, suggesting that agrin formed a complex with hMuSK and mouse Lrp4 and induced downstream signaling activation (Supplementary Fig. 5D). In our assay, the formation of AChR clustering was significantly suppressed in the presence of 5 out of 12 MuSK-MG sera at a concentration of 1% (Fig. 5, B and Supplementary Fig. 6). Although not statistically significant, the other seven sera (Patients No. 3–5, 7, 8, 10, and 12) also achieved some degree of suppression of AChR clustering. Most of the sera containing MuSK autoantibodies at a concentration of 20 nmol/mL or higher showed stronger suppression of AChR clustering, suggesting that the pathogenic capacity of sera from MuSK-MG patients observed in our assay was roughly correlated with the MuSK autoantibody concentration (Table 1). Interestingly, Ab#40-H significantly enhanced the formation of AChR clusters, even in the presence of the MuSK-MG sera from different patients (Fig. 5B and C and Supplementary Fig. 6). These results suggested that our MuSK agonistic antibody has the potential to restore MuSK signaling inhibited by MuSK autoantibodies and enhance AChR clustering in MuSK-MG patients, including those who do not respond to steroids.

### Ab#40-H activates MuSK signaling by binding to MuSK in the presence of autoantibody

To investigate the effects of our MuSK agonistic antibody on AChR clustering in the presence of a pathophysiological concentration of MuSK autoantibodies, we planned to conduct AChR clustering assay with C2C12 cells under conditions with a higher concentration of serum. However, a preliminary experiment using autoantibody-free pooled serum from healthy individuals showed that high concentrations of serum reduced AChR clustering in a concentration-dependent manner. These data suggest that serum-derived components may directly or indirectly interfere with the AChR clustering assay (Supplementary Fig. 7). To minimize this nonspecific effect, IgG from each serum sample of five MuSK-MG patients (No. 2, 3, 6, 7, and 12) was purified using a Protein A column and elution buffer was replaced with PBS. These samples were selected because serum purification required 500 μL of serum per patient, and the remaining serum volumes from other patients were insufficient for purification. The final total IgG concentration was adjusted to match that of the original serum, and more than 50% of total IgG and total IgG4, a subclass that includes MuSK-specific autoantibodies, was recovered after purification (Supplementary Fig. 8, A-C). While the purified IgG antibodies dose-dependently reduced the effect of Ab#40-H to induce AChR clustering, Ab#40-H significantly enhanced AChR clustering in the presence of 3 out of 5 patient-derived IgG samples, at a concentration equivalent to 10% serum IgG (Fig. 6A).

**Fig. 6:**
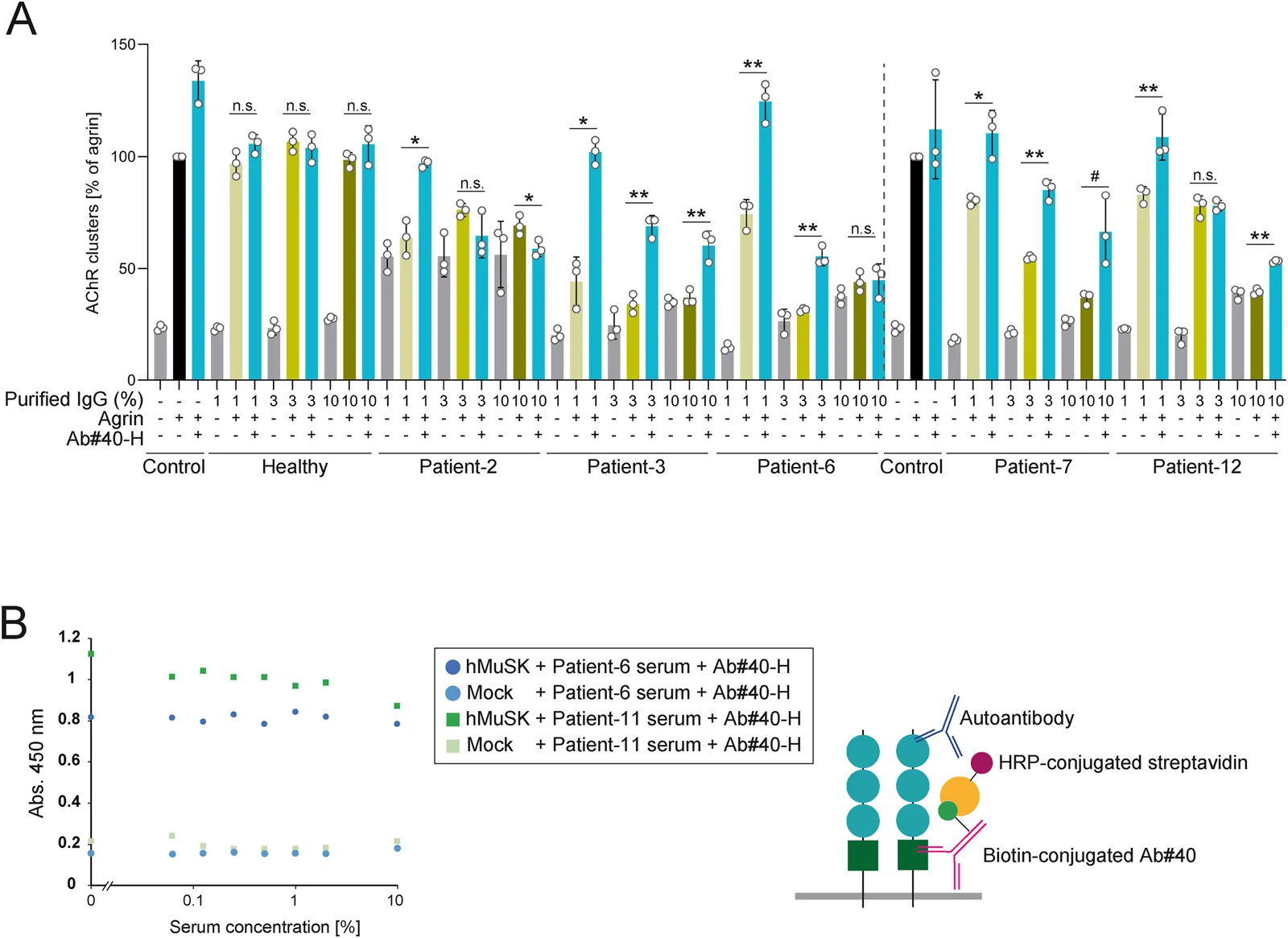
**High concentration of patient serum reduced Ab#40-H-induced AChR clustering without affecting the interaction between Ab#40-H and MuSK.**(**A**) Effect of Ab#40-H on AChR clustering in the C2C12 myotube expressing hMuSK was evaluated in the presence or absence of IgG antibodies purified from serum of each MG patient or control subject (Healthy). The bar graphs represent mean ± S.D. (n = 3, Welch's *t*-test between serum- and Ab#40-H-treated groups: ∗P < 0.05 and ∗∗P < 0.01, and n.s., no significance; or between 10% patient serum- and Ab#40-H-treated groups: Student's t-test #P < 0.05). (**B**) Competitive binding assays between Ab#40-H and autoantibodies in serum from patients with MuSK-MG (Patient-6 and Patient No-11) against hMuSK were performed. The binding levels of Ab#40-H to hMuSK were determined by the ABC-ELISA method (right). Schematic illustration represents the ABC-ELISA method (left).

We attempted to analyze the relationship between MuSK agonistic antibody and MuSK-MG autoantibodies from a molecular pathology perspective using sera from patients No. 6 and No. 11. A dose-dependent increase in autoantibody binding, within the range of 0%–10%, was detected by the cell ELISA using 293 A cells expressing hMuSK (Supplementary Fig. 9). Notably, the binding of autoantibodies did not affect the binding of biotin-labeled Ab#40-H to hMuSK, as detected by HRP-conjugated streptavidin (Fig. 6B). These findings indicated that patient-derived autoantibodies did not substantially compete with Ab#40-H for MuSK binding under the cell ELISA conditions used in this study, which partially supported the result that a 10% concentration of IgG from patient sera did not completely abolish the agonistic activity in the AChR clustering assay (Fig. 6A).

## Discussion

This study extends previous findings about MuSK-targeted therapy by demonstrating the efficacy of MuSK agonistic antibodies in both the active immunization MuSK-MG mouse model and the humanized myotube model in the presence of MuSK-MG patient autoantibodies. Ab#40-H and Ab#03-H, generated in this study, showed different KD values but both exhibited almost comparable MuSK agonistic activity in cell based assays. These antibodies share the IgG1 isotype and the same target domain as the previously reported MuSK agonist ARGX-119 [[18], [19], [20], [21], [22]]. Although side-by-side functional comparison with ARGX-119 was not possible because ARGX-119 and other previously reported agonistic antibodies were not available to us, our antibodies induced activation of MuSK signaling *in vitro* and demonstrated efficacy in a MuSK-MG model distinct from the previously reported passive-transfer model. Taken together, these findings further support pharmacological activation of MuSK as a promising therapeutic approach for MuSK-MG.

Our MuSK agonistic antibodies induced full activation of the MuSK signaling pathway through binding to the Fz domain, similar to those reported previously [[18], [19], [20], [21], [22]]. In consideration of the finding that Wnt-9a and Wnt-11 directly bind to the Fz domain of MuSK and activate its downstream signaling in an LRP4-dependent and agrin-independent manner [31], our data evidently support the hypothesis that the Fz domain of MuSK plays a key role to inducing MuSK homodimerization and activating its downstream signaling pathway. Utilizing these antibodies and a humanized myotube model, we precisely evaluated the effects of patient-derived autoantibodies on agonistic antibody-mediated MuSK signaling recovery. At first, we confirmed that 5 out of 12 sera significantly inhibited the agrin-induced AChR clustering at the serum concentration of 1%. Notably, treatment with MuSK agonistic antibody significantly reversed the autoantibody-mediated inhibition of MuSK signaling, achieving a signal level comparable to that induced by agrin. Those sera originated from patients showing different disease severity including severe myasthenic symptoms as defined by the MGFA classification, and different steroid responses including non-responders. These findings indicate that a MuSK agonistic antibody may show efficacy in most patients with MuSK-MG. However, it should also be noted that the MuSK signaling recovery induced by the agonistic antibody tended to decrease when the purified IgG concentration was increased to 10%.

While the blood concentration of anti-MuSK autoantibodies is measurable, the distribution of these autoantibodies within muscle tissue, particularly within NMJs, remains to be elucidated. As indicated by the pharmacokinetic study, 34%–35% of injected therapeutic antibodies in the bloodstream are transferred into muscle tissue in mice [32]. Moreover, a physiologically based pharmacokinetic analysis of antibodies showed that 3.97% of the antibodies present in the plasma were distributed in human muscle tissue [33]. These data indicate that the transfer of serum IgG into muscle tissue may exceed 3%. The 3% and 10% serum concentrations used in the *in vitro* experiments are thus physiologically relevant to human muscle tissue. As there was no direct competition between the autoantibody and the agonistic antibody at 10% serum concentration, the observed decrease in MuSK signaling recovery by the MuSK agonistic antibodies could not be explained by competitive inhibition. From recent studies, it has become clear that MuSK autoantibodies in patients with MuSK-MG include various types, such as bispecific type (Fab-arm exchange) and kappa/lambda chain type [30,[34], [35], [36], [37]]. IgG1–3 MuSK autoantibodies can impair AChR cluster formation through a noncanonical mechanism that does not inhibit MuSK, DOK7, or βAChR phosphorylation and can be restored by an SHP2 inhibitor [38]. Certain types of these autoantibodies may interfere with the effects of the MuSK agonistic antibody on MuSK signaling recovery. Further studies using MuSK-MG animal models with varying levels or types of autoantibodies will provide deeper insights into the effects of autoantibodies on the therapeutic efficacy of a MuSK-activating antibody. Of note that, a limitation of the purified-IgG assay is that this purification removes non-IgG serum components and therefore does not fully reproduce the physiological serum environment. Thus, factors that may influence IgG4 behavior, including dynamic Fab-arm exchange, may not be completely preserved in this assay. In addition, total IgG4 recovery was used only as an indirect indicator of recovery of the IgG4 fraction and does not directly quantify MuSK-specific IgG4. Therefore, the 1% whole-serum assay and the 10% purified-IgG assay should be interpreted as complementary experimental systems rather than directly equivalent conditions.

Generalized MG (gMG) including MuSK-MG is an autoimmune disease with a high unmet medical need [4]. The first-line treatment is usually steroids, but in most patients the disease is not sufficiently controlled by steroids alone. Moreover, even those who respond to steroids often suffer from adverse effects of long-term steroid use, resulting in a decreased quality of life [4]. It is thus important to develop effective therapies addressing the pathogenic mechanisms of MuSK-MG. Recently, anti-C5 antibodies (e.g., eculizumab), which block the formation of the membrane attack complex (MAC), have been approved for the treatment of anti-AChR antibody-positive gMG patients (AChR-MG) [39]. The collapse of NMJs caused by MAC formation is the critical pathogenic mechanism of AChR-MG and thus suppression of the complement pathway is a reasonable approach for treating this disease [40,41]. In contrast, autoantibodies from MuSK-MG patients are of the IgG4 subclass and do not induce MAC formation. Therefore, for MuSK-MG patients, MuSK activation, rather than complement pathway inhibition, is an effective therapeutic approach. In some studies, rituximab also showed efficacy for gMG. Rituximab is considered to show efficacy through the recovery of MuSK signaling by reducing the MuSK antibody level by depleting CD20^+^ B cells [42,43]. Efgartigimod, which is a neonatal Fc receptor antagonist and decreases autoantibody levels, is approved for gMG in several countries [44,45]. However, long-term use of these immune system modulators increases the risk of infections [46,47]. Therefore, direct activation of MuSK by a MuSK agonistic antibody, which does not affect the immune system, may likely to be a safer and more effective approach for the lifelong treatment of gMG [19,22]. Although the result of the AChR clustering assay in this study implies that MuSK autoantibodies may impede the full activation of MuSK signaling by an agonistic antibody, a status of minimal manifestations or better may be achieved by supplemental use of steroid or immunosuppressant. It is expected that such combination therapy would help to minimize the dose of steroid or immunosuppressive agent required to achieve a status of minimal manifestations or better, which would contribute to reducing the risk of side effects associated with polytherapy of immunosuppressants. Furthermore, given the potential for decreased efficacy due to interference by certain autoantibodies, the stratification of MuSK-MG patients according to their autoantibody type may be crucial for the identification of those who will respond well to treatment with MuSK agonist antibodies. In this context, “autoantibody type” refers to molecular and functional characteristics of MuSK autoantibodies, including IgG subclass composition, the proportion of Fab-arm-exchanged IgG4, κ/λ light-chain usage. To further validate this therapeutic concept, future studies should include side-by-side comparisons between MuSK agonistic antibodies and established immunotherapies, such as rituximab, using appropriately designed experimental models.

The activation of MuSK signaling with agonist antibodies may be a promising therapeutic approach not only for MuSK-MG, but also for other diseases with NMJ dysfunction caused by diminished MuSK signaling, such as CMS and sarcopenia. The efficacy of MuSK agonistic antibodies in improving NMJ formation in DOK-7-deficient CMS mice was reported, suggesting that certain types of CMS (e.g., Agrin-CMS) are candidates for treatment with MuSK agonistic antibodies [18]. Age-related sarcopenia, in which reduction of MuSK signaling is observed, is another of these diseases [48]. Forced activation of MuSK by AAV-DOK-7 in aged mice showed significant improvements in motor function and muscle strength with innervation of the NMJs [49,50]. In particular, the potential risks of efficacy reduction observed in this study are no longer a concern, since these diseases are not caused by autoantibodies to MuSK. Therefore, age-related sarcopenia and CMS are candidates for the application of MuSK-activating antibodies for therapeutic purposes.

Our study has some limitations. First, the pathophysiology of the MuSK-MG model may not fully replicate that of patients with MuSK-MG. In our disease model, MuSK Ig1 peptide immunization was associated with an increase in total IgG2a level. While mouse IgG2a has higher complement-dependent cytotoxicity (CDC) activity, the predominant autoantibody subclass in patients with MuSK-MG is human IgG4, which does not induce CDC [11]. The influences of complement-mediated injury on the NMJ remain uncertain because MuSK-specific IgG subclasses were not directly measured in this study. Antigen-specific subclass assays will be required to clarify the subclass composition of MuSK-specific autoantibodies in this model. In addition, mice do not have an antibody subclass that undergoes Fab-arm exchange equivalent to human IgG4 [51]. Because MuSK antibody effects on MuSK signaling can depend on antibody valency, epitope specificity, and antibody format and the composition of the antibody repertoire, these differences may affect the translatability of the active immunization model. Furthermore, we did not directly confirm the binding of Ab#03-H to mMuSK at NMJs *in vivo* by immunohistochemistry. Further studies using passive-transfer models with functionally monovalent MuSK antibodies will be required for translational validation. Second, the mechanism underlying the reduction of agonistic activity by higher concentrations of autoantibodies remains unclear. Our AChR clustering assay showed that high concentrations of autoantibodies reduced the effect of the agonistic antibody to induce AChR clustering, while the competitive binding assay did not provide evidence of competitive inhibition between agonistic antibodies and autoantibodies in our assay condition. To clarify which serum components or IgG characteristics determine the functional outcome of the agonistic antibody, further investigation is needed using a passive-transfer model of MuSK-MG developed with different sera from patients with MuSK-MG and more detailed kinetic analyses by SPR to assess the binding properties of patient-derived autoantibodies and agonistic antibodies to MuSK. Third, outcome measures such as wire-hanging latency, EMG analysis, and imaging-based quantification may have been influenced by unconscious bias, because the efficacy analyses were not performed under blinded conditions. Because the active immunization MuSK-MG model used in this study showed a relatively mild disease phenotype, variation in serum anti-MuSK antibody titers among groups, including the lower mean relative titer observed in the Ab#03-H treated group, may have contributed to the measured differences in phenotype. As mean AChR fluorescence intensity was quantified only demonstrating equivalent disease severity, antibody responses, within threshold-defined AChR-positive regions, relatively dim signals below the threshold might be excluded, and the measurement may not directly reflect total AChR content. The results should not be interpreted as pharmacokinetics, or therapeutic responsiveness between these two strains, because the efficacy experiments using A/J and C57BL/6 J mice were conducted independently and were not designed as a side-by-side comparison of strain-dependent differences. In addition, the relative serum anti-MuSK IgG titers should be interpreted with caution. Although the ELISA was designed to detect mouse anti-MuSK IgG using recombinant rat MuSK-coated plates and an anti-mouse IgG as secondary antibody, we did not directly test whether Ab#03-H binds to immobilized rat MuSK or interferes with endogenous mouse anti-MuSK IgG binding. Thus, possible assay interference cannot be excluded, and the lower apparent titers observed in Ab#03-H-treated groups may not necessarily reflect a true reduction in endogenous anti-MuSK IgG levels. Lastly, the clinical advantages of MuSK agonistic antibodies compared to RTX and efgartigimod in patients with MuSK-MG are still undefined, as our antibody does not have the ability to reduce autoantibody levels.

In conclusion, our results in this study provide valuable insights into the use of a MuSK agonistic antibody as a novel treatment for patients with MuSK-MG. The findings indicate that the efficacy of the MuSK agonistic antibody is susceptible to variation in patients’ autoantibody profile, including blood levels and types. Therefore, selecting good responders among MuSK-MG patients and considering add-on therapies to minimize immunosuppressant use represent promising strategies for the effective clinical use of the MuSK agonistic antibody in the future.

## Author contributions

K.Ya., R.N., G.N., and T.N. conceptualized the study; K.Ya., R.N., and G.N. designed the experiments; K.Yo., G.N., M.S., C.Y., and Y.Na. developed the antibodies; K.Ya., G.N., Y.Ne., M.Yam., H.K., and R.N. performed the experiments; K.Ya. and H.T. made the illustrations; K.Ya., T.N., K.T., A.U., and S.K. were responsible for administration of the project; T.N., A.U., S.K., and K.T. supervised the project; K.Ya., H.T., and A.U. wrote the original draft of the manuscript; and K.Ya., H.T., T.N., A.U., M.Yas., H.A., Y.O., H.H., E.O., and S.K. reviewed and edited its final draft. All authors approved the final version of the manuscript.

## Ethics approval and consent for participation

All human derived samples were obtained with written informed consent to participate, and this study was approved by Chiba University (No. 3624) and Daiichi Sankyo Co., Ltd. (No. 000359–04).

## Data and materials availability

All data associated with this study are present in the paper or the Supplementary Materials.

## Funding sources

This study was fully financially supported by 10.13039/501100002973Daiichi Sankyo Co., Ltd.

## Declaration of competing interest

The authors declare that they have no known competing financial interests or personal relationships that could have appeared to influence the work reported in this paper.The authors declare the following financial interests/personal relationships which may be considered as potential competing interests:
