# Supplementary material for: MuSK agonistic antibody has therapeutic potential for MuSK antibody-positive myasthenia gravis
Source: Neurotherapeutics. 2026 Oct 5;23(6):e01090. doi: 10.1016/j.neurot.2026.e01090 (PMC13673047; doi:10.1016/j.neurot.2026.e01090)
Supplement: Supplementary file 1 [file mmc10.pdf]

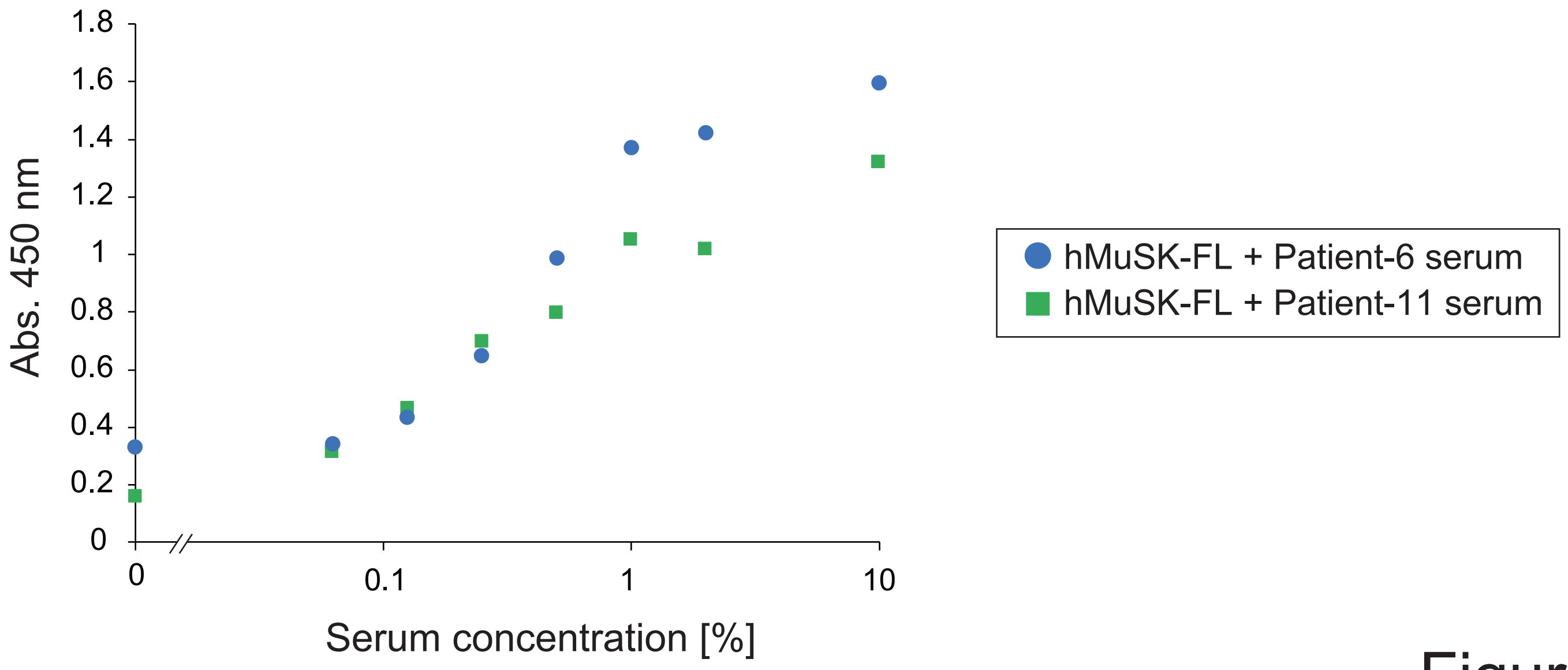

1.8
1.6
1.4
1.2
1
0.8
0.6
0.4
0.2
0
Abs. 450 nm
0
0.1
1
10
Serum concentration [%]
hMuSK-FL + Patient-6 serum
hMuSK-FL + Patient-11 serum

Figure S9
