# Supplementary material for: MuSK agonistic antibody has therapeutic potential for MuSK antibody-positive myasthenia gravis
Source: Neurotherapeutics. 2026 Oct 5;23(6):e01090. doi: 10.1016/j.neurot.2026.e01090 (PMC13673047; doi:10.1016/j.neurot.2026.e01090)
Supplement: Supplementary file 2 [file mmc2.pdf]

A

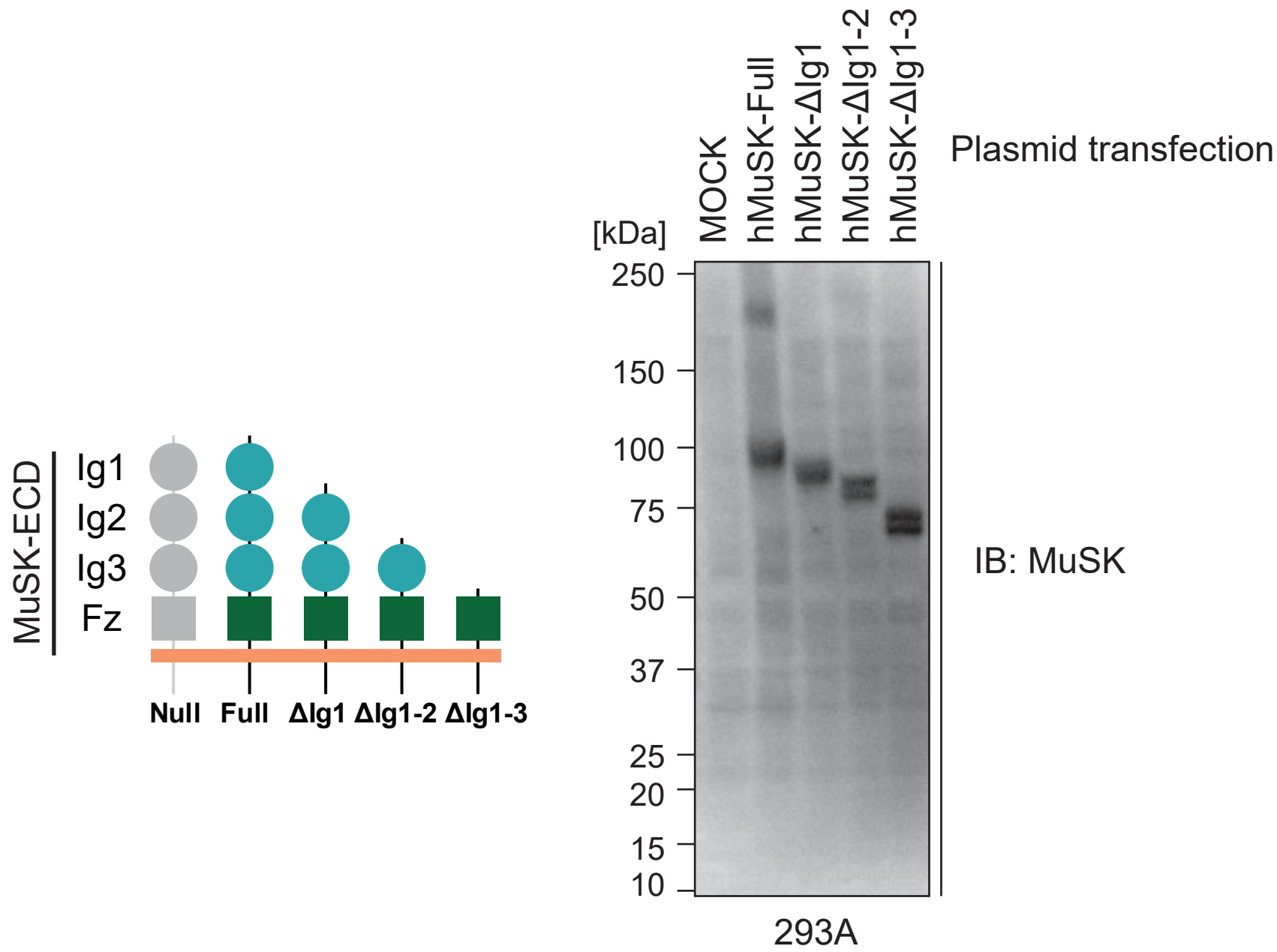

Construct	Mean intensity	Background-subtracted intensity	Relative expression
Full	41470.27	14394.42	1.03
ΔIg1	41005.19	13929.34	1.00
ΔIg1-Ig2	41639.6	14563.75	1.05
ΔIg1-Ig3	42369.07	15293.22	1.10
Background	27075.85	0	

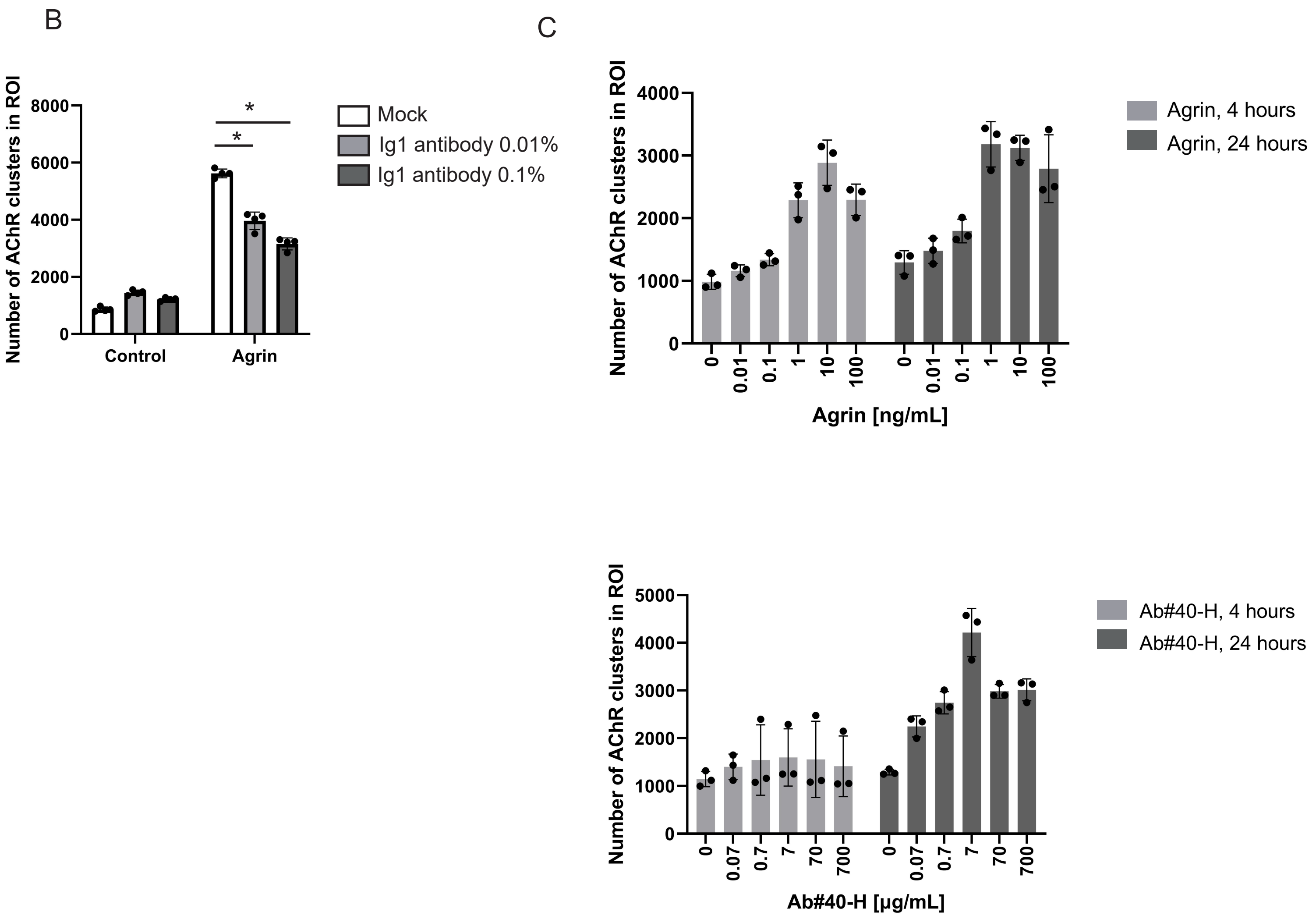

Figure S1
