# Supplementary material for: MuSK agonistic antibody has therapeutic potential for MuSK antibody-positive myasthenia gravis
Source: Neurotherapeutics. 2026 Oct 5;23(6):e01090. doi: 10.1016/j.neurot.2026.e01090 (PMC13673047; doi:10.1016/j.neurot.2026.e01090)
Supplement: Supplementary file 3 [file mmc3.pdf]

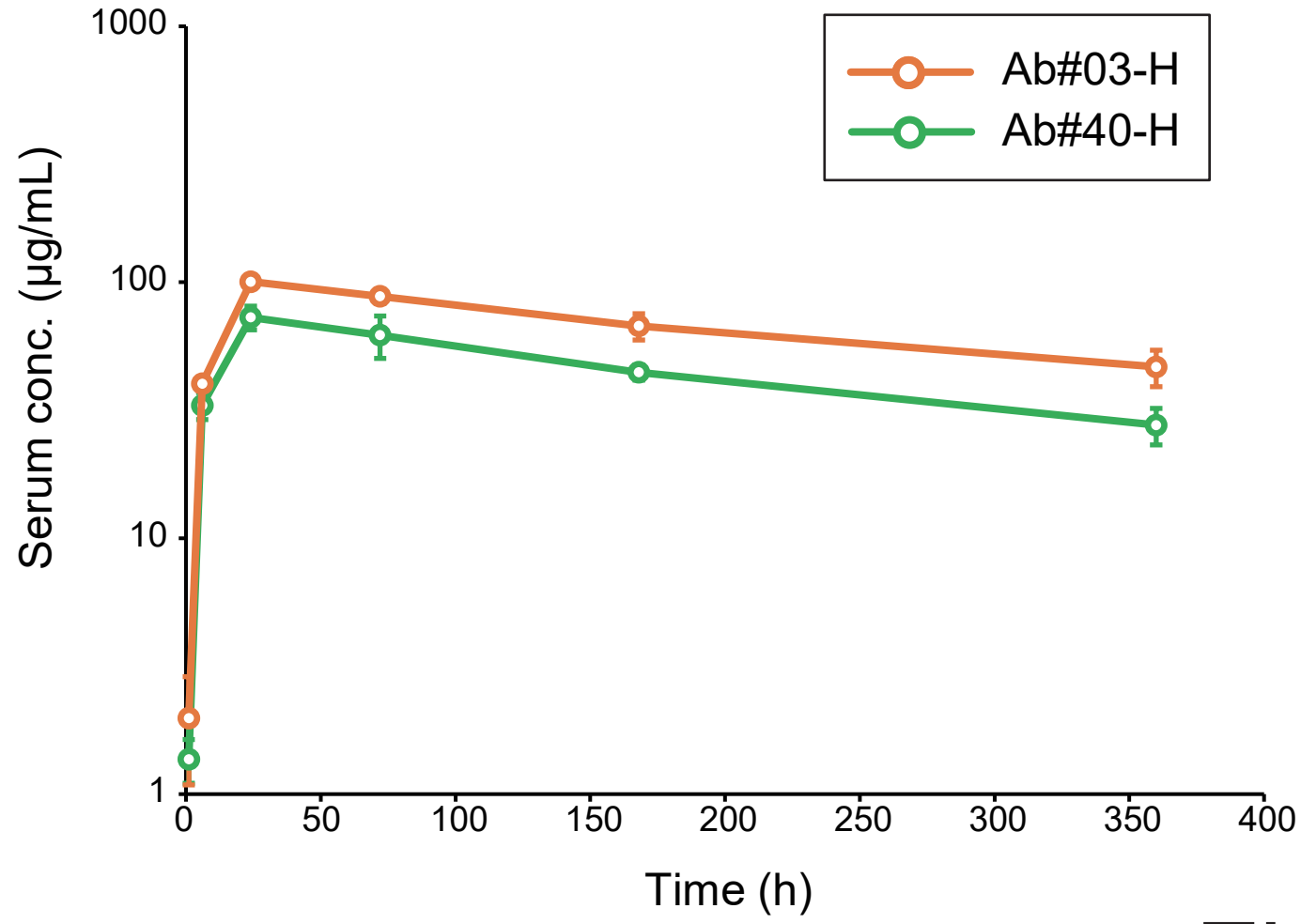
Ab#03-H
Ab#40-H
Serum conc. (μg/mL)
1000
100
10
1
0
50
100
150
200
250
300
350
400
Time (h)

Figure S2
