# Supplementary material for: MuSK agonistic antibody has therapeutic potential for MuSK antibody-positive myasthenia gravis
Source: Neurotherapeutics. 2026 Oct 5;23(6):e01090. doi: 10.1016/j.neurot.2026.e01090 (PMC13673047; doi:10.1016/j.neurot.2026.e01090)
Supplement: Supplementary file 4 [file mmc4.pdf]

Quantification of total serum levels of each mouse IgG subclass (IgG1, IgG2a, IgG2b, and IgG3)

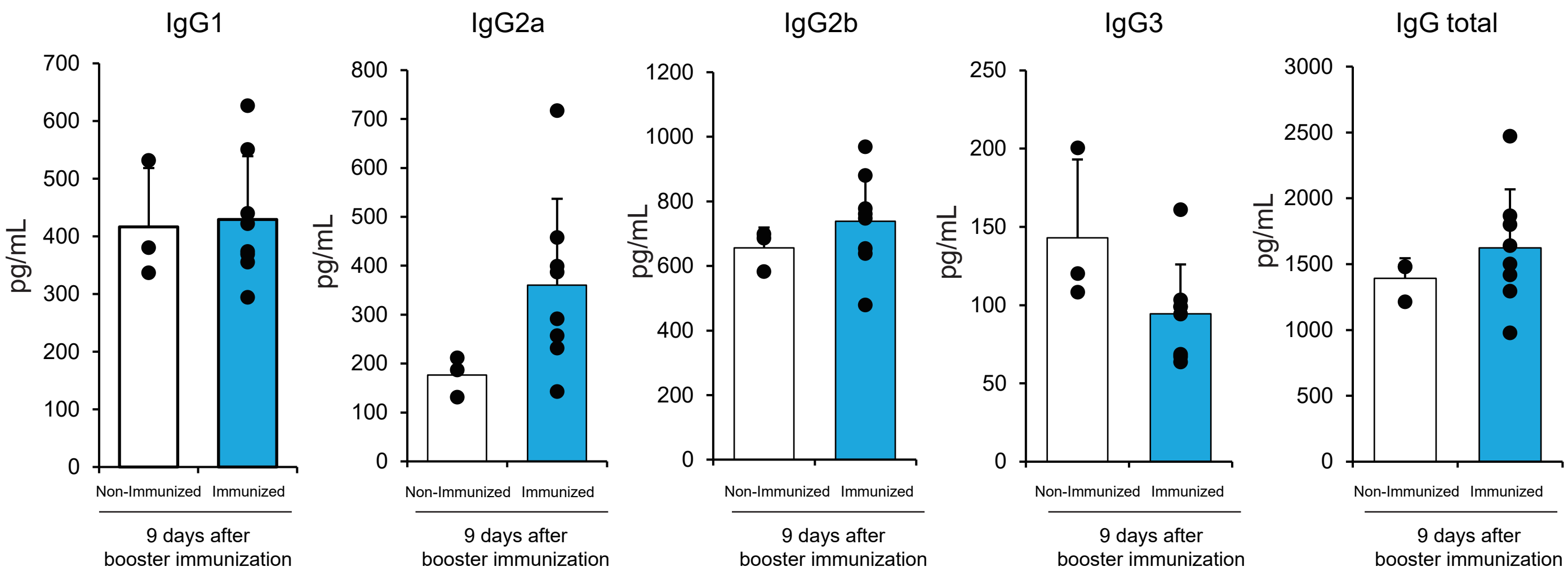

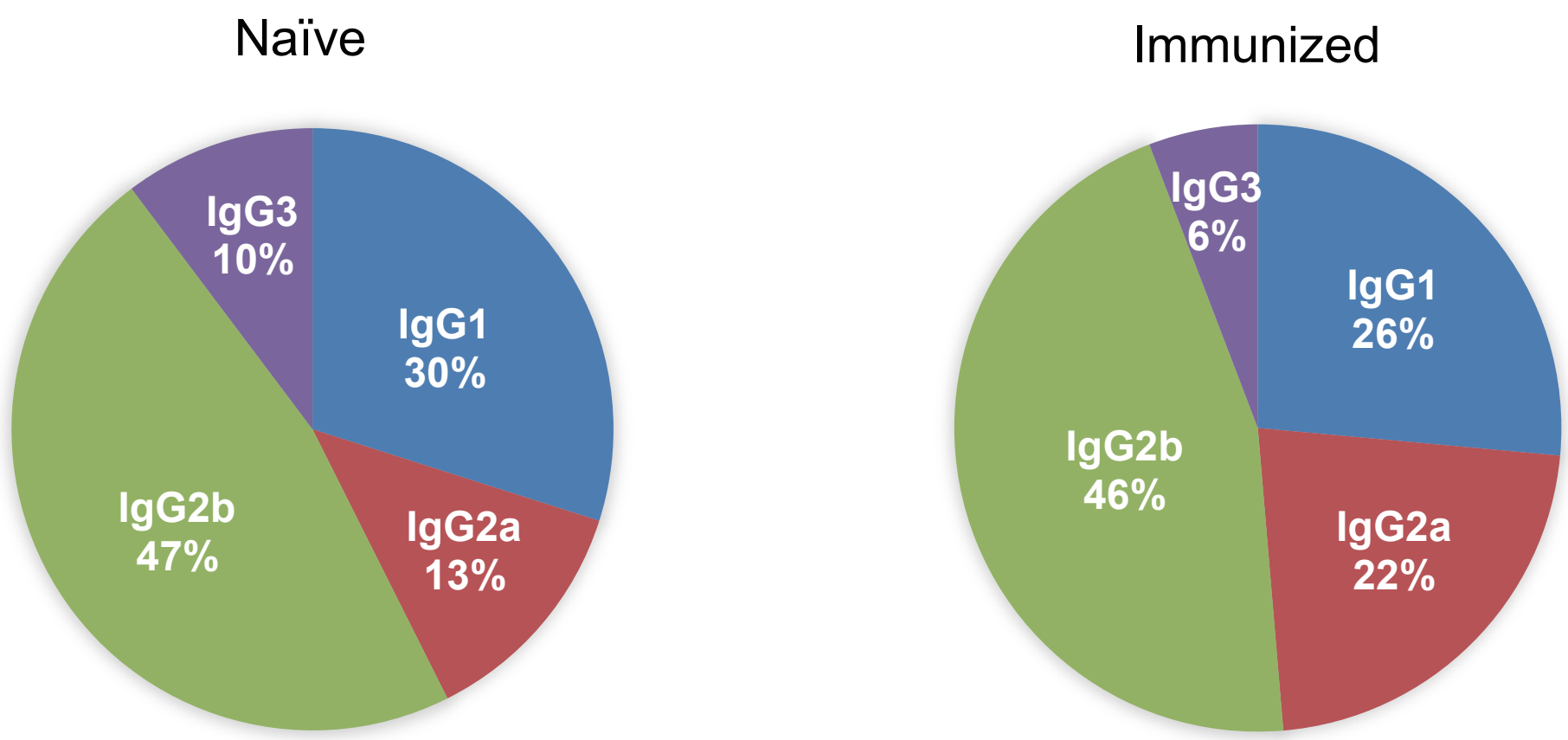

Figure S3
