# Supplementary material for: MuSK agonistic antibody has therapeutic potential for MuSK antibody-positive myasthenia gravis
Source: Neurotherapeutics. 2026 Oct 5;23(6):e01090. doi: 10.1016/j.neurot.2026.e01090 (PMC13673047; doi:10.1016/j.neurot.2026.e01090)
Supplement: Supplementary file 5 [file mmc5.pdf]

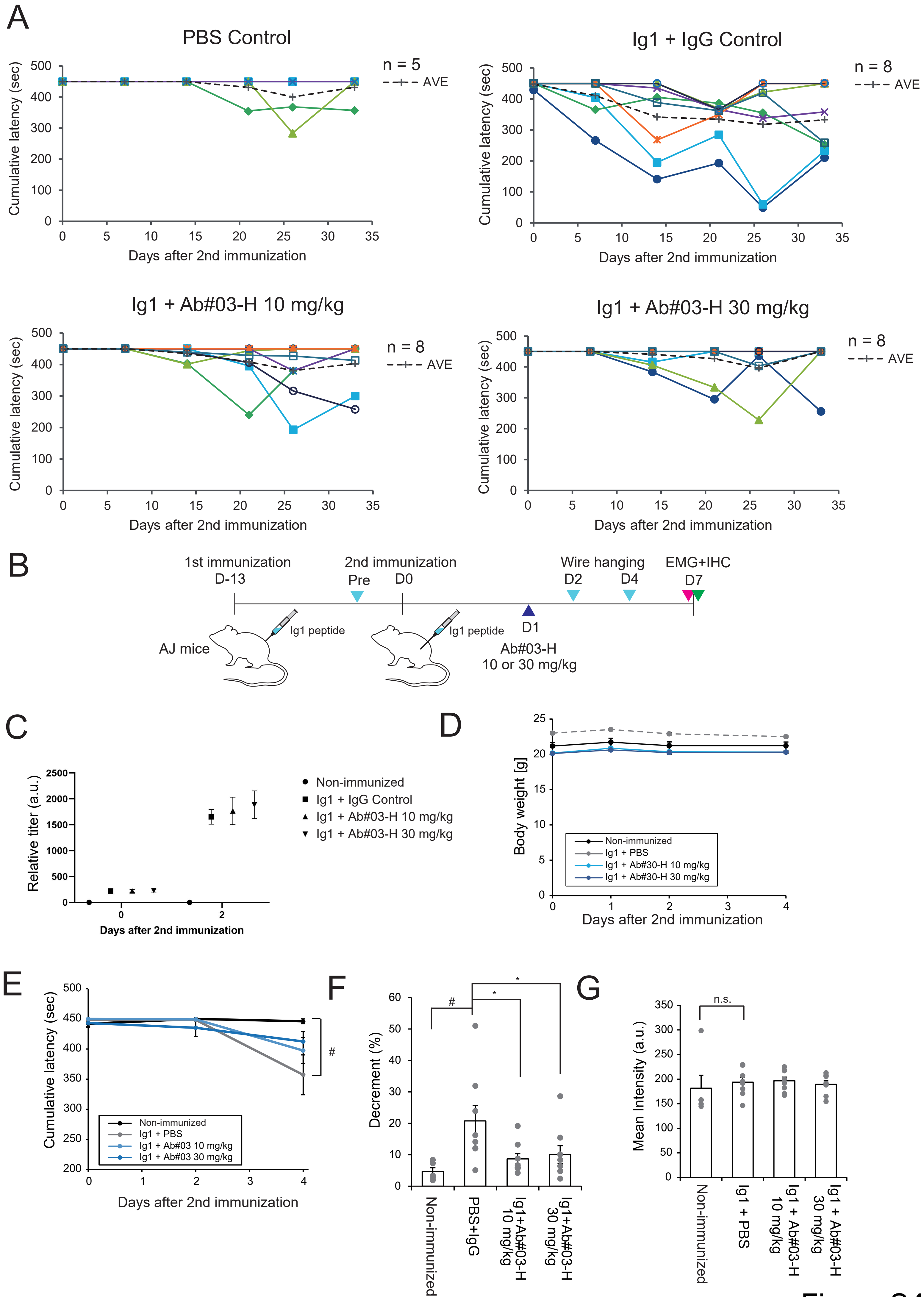
A
PBS Control
n = 5
AVE
Cumulative latency (sec)
Days after 2nd immunization
Ig1 + IgG Control
n = 8
AVE
Ig1 + Ab#03-H 10 mg/kg
n = 8
AVE
Ig1 + Ab#03-H 30 mg/kg
n = 8
AVE
B
1st immunization
D-13
2nd immunization
Pre
D0
Wire hanging
D2
D4
EMG+IHC
D7
AJ mice
Ig1 peptide
Ig1 peptide
D1
Ab#03-H
10 or 30 mg/kg
C
Relative titer (a.u.)
Days after 2nd immunization
Non-immunized
Ig1 + IgG Control
Ig1 + Ab#03-H 10 mg/kg
Ig1 + Ab#03-H 30 mg/kg
D
Body weight [g]
Non-immunized
Ig1 + PBS
Ig1 + Ab#30-H 10 mg/kg
Ig1 + Ab#30-H 30 mg/kg
Days after 2nd immunization
E
Cumulative latency (sec)
Non-immunized
Ig1 + PBS
Ig1 + Ab#03 10 mg/kg
Ig1 + Ab#03 30 mg/kg
#
Days after 2nd immunization
F
Decrement (%)
#
*
*
Non-immunized
PBS+IgG
Ig1+Ab#03-H 10 mg/kg
Ig1+Ab#03-H 30 mg/kg
G
Mean Intensity (a.u.)
n.s.
Non-immunized
Ig1 + PBS
Ig1 + Ab#03-H 10 mg/kg
Ig1 + Ab#03-H 30 mg/kg

Figure.S4
