# Supplementary material for: MuSK agonistic antibody has therapeutic potential for MuSK antibody-positive myasthenia gravis
Source: Neurotherapeutics. 2026 Oct 5;23(6):e01090. doi: 10.1016/j.neurot.2026.e01090 (PMC13673047; doi:10.1016/j.neurot.2026.e01090)
Supplement: Supplementary file 6 [file mmc6.pdf]

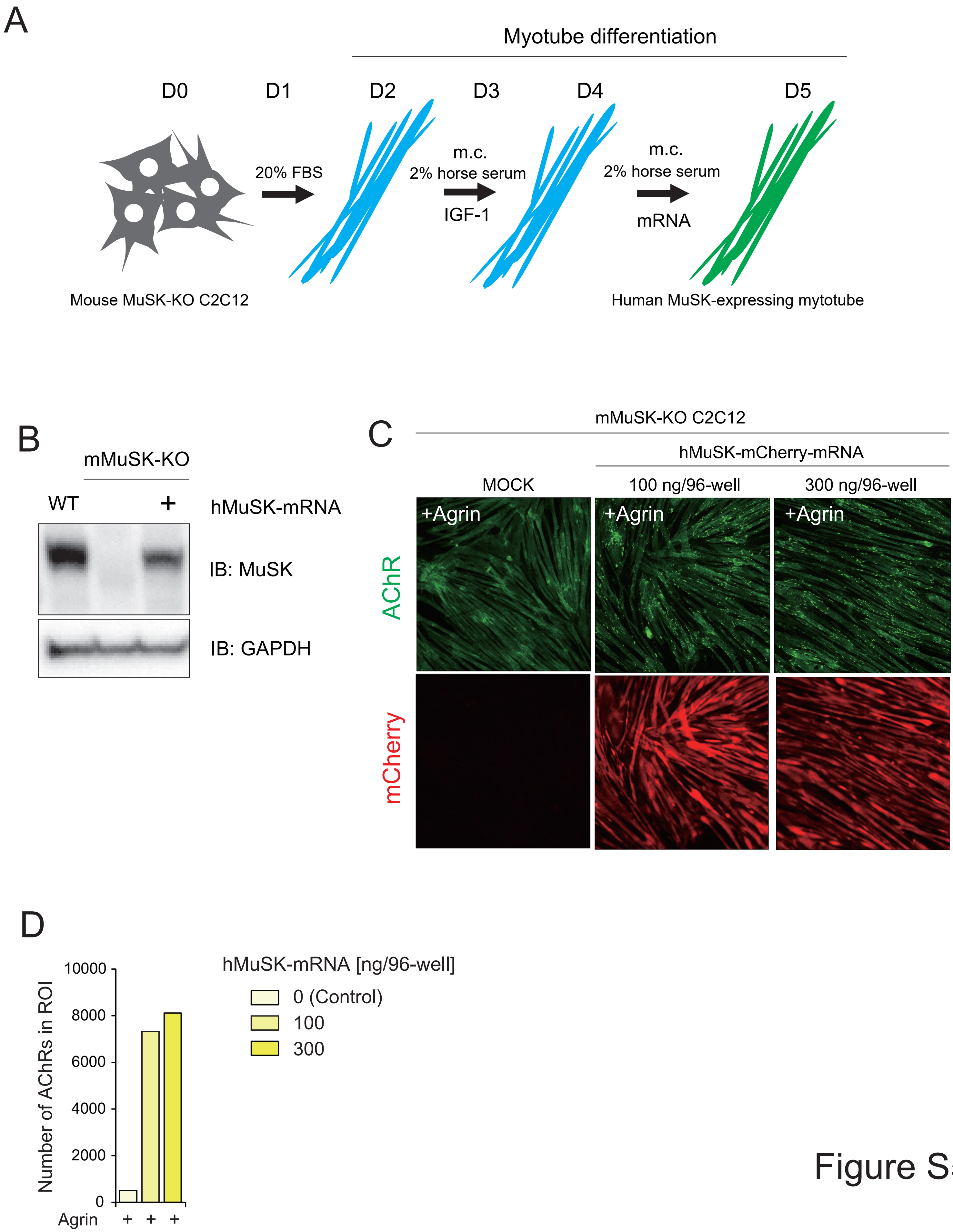
A
Myotube differentiation
D0
D1
D2
D3
D4
D5
20% FBS
m.c.
2% horse serum
IGF-1
m.c.
2% horse serum
mRNA
Mouse MuSK-KO C2C12
Human MuSK-expressing mytotube
B
mMuSK-KO
WT
+
hMuSK-mRNA
IB: MuSK
IB: GAPDH
C
mMuSK-KO C2C12
hMuSK-mCherry-mRNA
MOCK
100 ng/96-well
300 ng/96-well
+Agrin
+Agrin
+Agrin
AChR
mCherry
D
hMuSK-mRNA [ng/96-well]
0 (Control)
100
300
Number of AChRs in ROI
10000
8000
6000
4000
2000
0
Agrin
+
+
+

Figure S5
