# Supplementary material for: MuSK agonistic antibody has therapeutic potential for MuSK antibody-positive myasthenia gravis
Source: Neurotherapeutics. 2026 Oct 5;23(6):e01090. doi: 10.1016/j.neurot.2026.e01090 (PMC13673047; doi:10.1016/j.neurot.2026.e01090)
Supplement: Supplementary file 7 [file mmc7.pdf]

A

AChR cluster (% of agrin)	MuSK autoantibody, nmol/mL	Agrin + serum	Agrin + serum + Ab#40-H (7 μg/mL)	Agrin + serum + Ab#40-H (70 μg/mL)
MOCK	—	100	173.3 (*)	170.8 (*)
Patient-1	21	68.8 (#)	115.7 (*)	127.6 (*)
patient-2	70	66.8 (#)	106.3 (*)	108.8 (*)
Patient-3	73	82	141.5 (*)	141.9 (*)
Patient-4	4.1	96.7	178.3 (*)	189.4 (*)
Patient-5	16.8	73.2	138.4 (*)	133.5 (*)

AChR cluster (% of agrin)	MuSK autoantibody, nmol/mL	Agrin + serum	Agrin + serum + Ab#40-H (0.7 μg/mL)	Agrin + serum + Ab#40-H (7 μg/mL)
MOCK	—	100	119.5	149.7(*)
Patient-6	36.9	77.2(#)	111.9(*)	125.7(*)
Patient-7	14.6	80	109.4	125.2(*)
Patient-8	6.9	83.7	110.5(*)	104.7(*)
Patient-9	54	78.8(#)	115.7(*)	112.1(*)

AChR cluster (% of agrin)	MuSK autoantibody, nmol/mL	Agrin + serum	Agrin + serum + Ab#40-H (0.7 μg/mL)	Agrin + serum + Ab#40-H (7 μg/mL)
MOCK	—	100	121.9	122.4
Patient-10	0.02	103	138.8(*)	135.2(*)
Patient-11	92	60.4(#)	79.1	85.3(*)
Patient-12	7.6	92	117.2	116.8

B

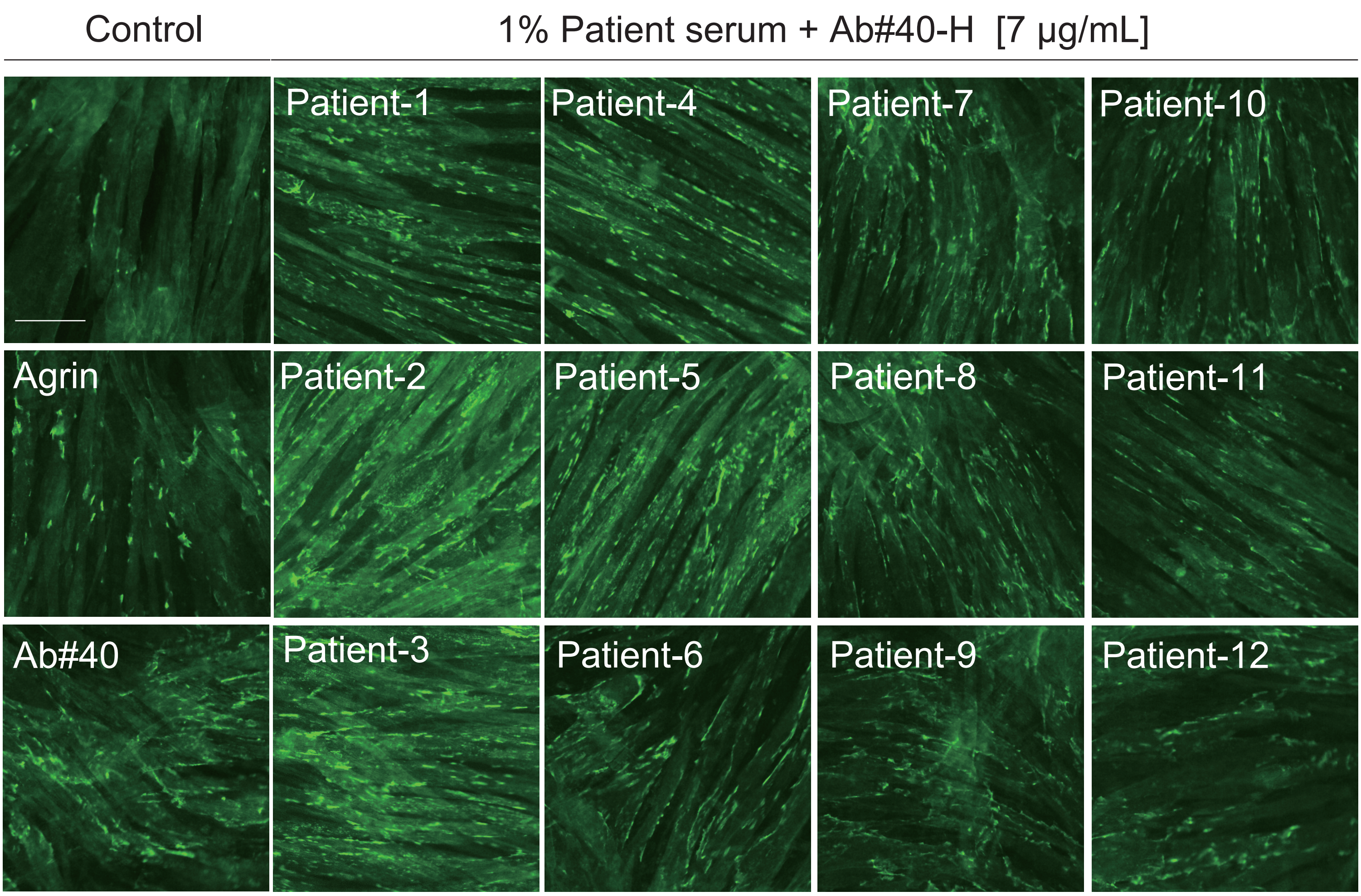

Figure S6
