# Supplementary material for: MuSK agonistic antibody has therapeutic potential for MuSK antibody-positive myasthenia gravis
Source: Neurotherapeutics. 2026 Oct 5;23(6):e01090. doi: 10.1016/j.neurot.2026.e01090 (PMC13673047; doi:10.1016/j.neurot.2026.e01090)
Supplement: Supplementary file 8 [file mmc8.pdf]

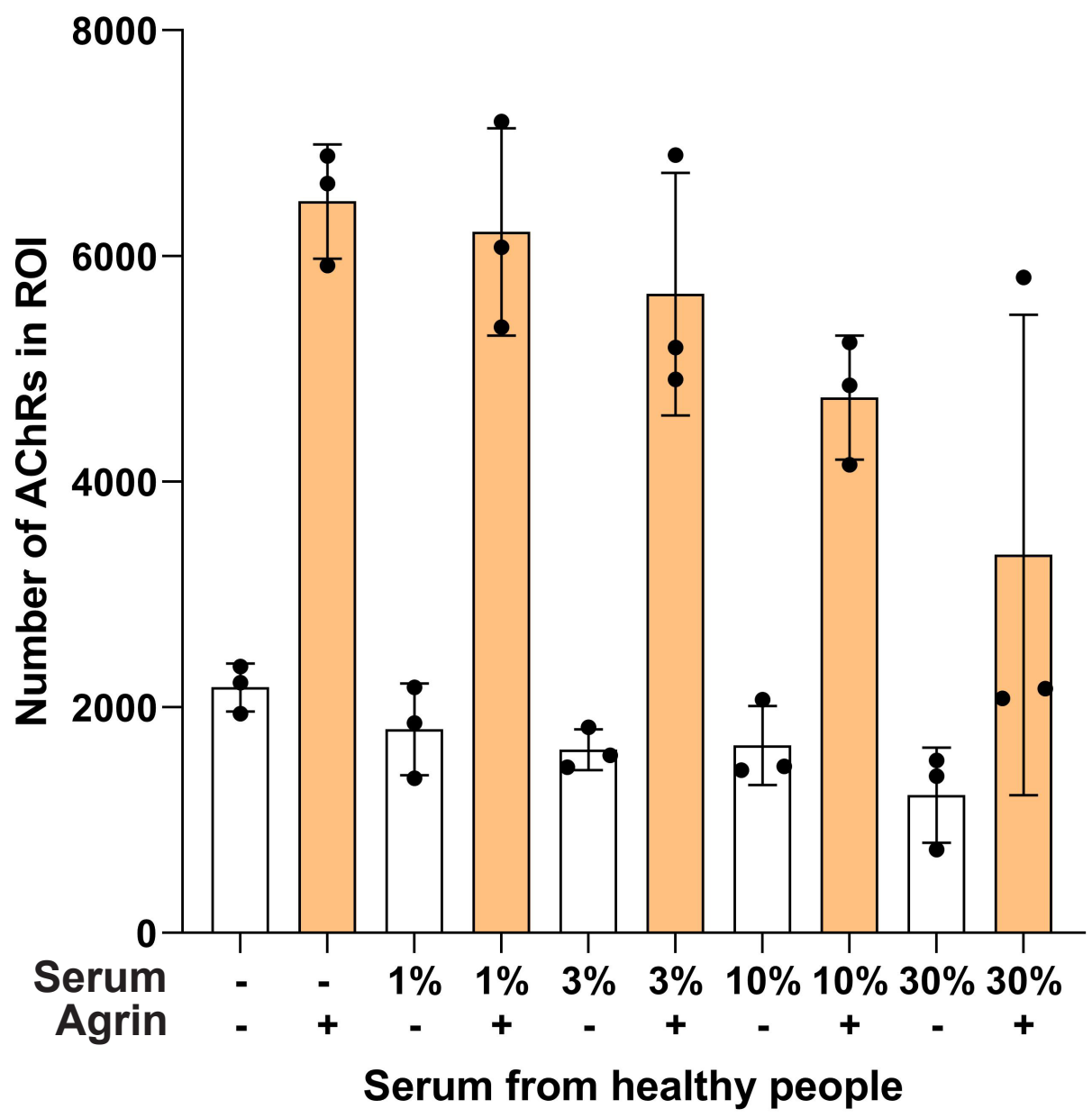
8000
6000
4000
2000
0
Number of AChRs in ROI
Serum - - 1% 1% 3% 3% 10% 10% 30% 30%
Agrin - + - + - + - + - +
Serum from healthy people

Figure S7
