# Supplementary material for: MuSK agonistic antibody has therapeutic potential for MuSK antibody-positive myasthenia gravis
Source: Neurotherapeutics. 2026 Oct 5;23(6):e01090. doi: 10.1016/j.neurot.2026.e01090 (PMC13673047; doi:10.1016/j.neurot.2026.e01090)
Supplement: Supplementary file 9 [file mmc9.pdf]

A

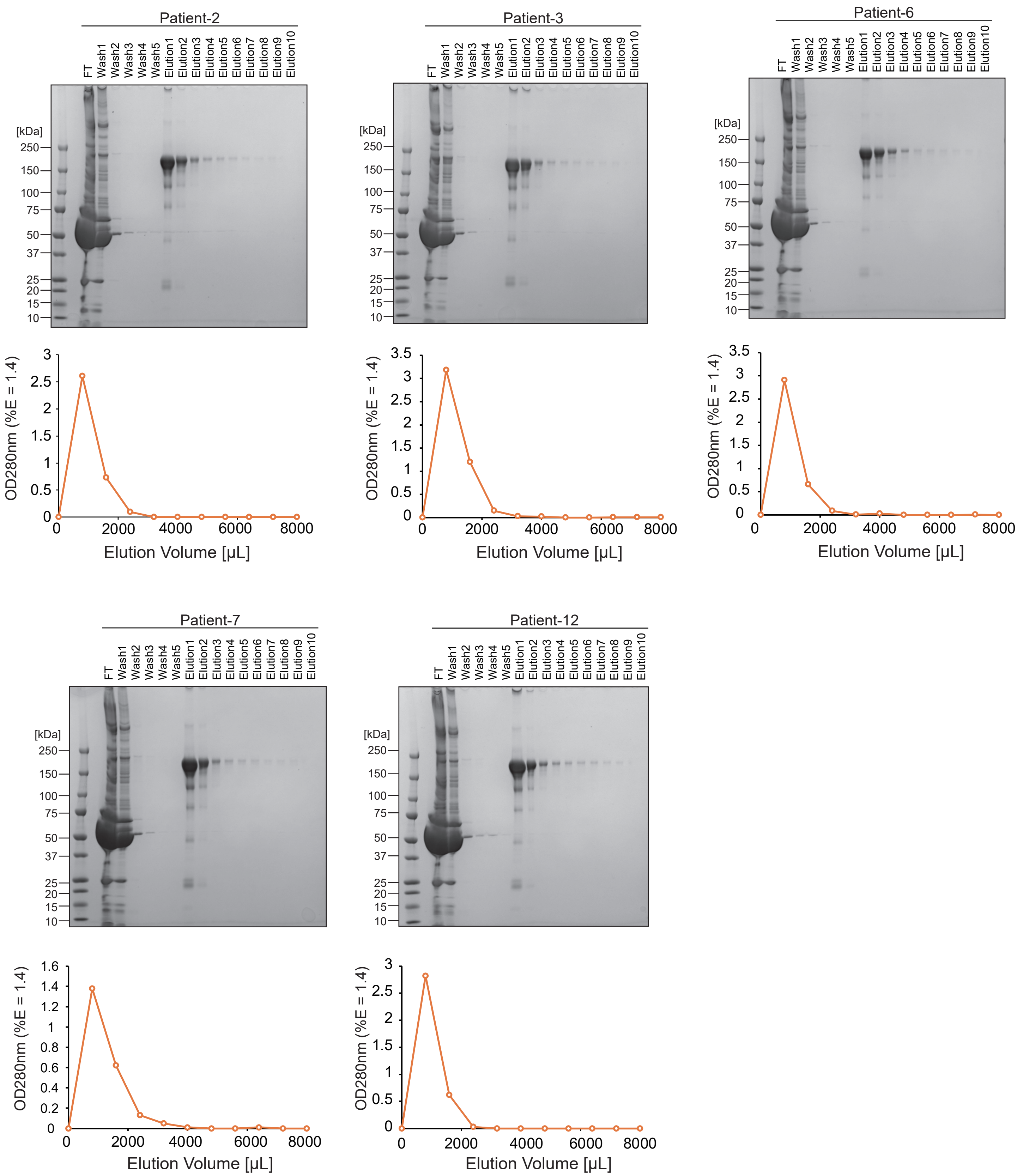

B

	Input (500 µL)	Purified product	Yield
Serum	Total IgG [µg]	Total IgG [µg]	[%]
Patient-2	3862.5	2048.4	53.0
Patient-3	4822.6	5831.9	120.9
Patient-6	4306.1	3116.8	72.4
Patient-7	3185.7	1946.0	61.1
Patient-12	4819.2	5151.7	106.9

C

	Input (500 µL)	Purified product	Yield
Serum	IgG4 [µg]	IgG4 [µg]	[%]
Patient-2	106	64	60
Patient-3	277	603	218
Patient-6	74	79	106
Patient-7	22	12	53
Patient-12	14	21	151

Figure S8
