# Supplementary material for: MuSK agonistic antibody has therapeutic potential for MuSK antibody-positive myasthenia gravis
Source: Neurotherapeutics. 2026 Oct 5;23(6):e01090. doi: 10.1016/j.neurot.2026.e01090 (PMC13673047; doi:10.1016/j.neurot.2026.e01090)
Supplement: Supplementary file 10 [file mmc1.docx]

**Supporting Information for**

MuSK agonistic antibody has therapeutic potential for MuSK antibody-positive myasthenia gravis

Kentaro Yamamoto^1^, Akiyuki Uzawa^2^, Genta Narazaki^1^, Yoshikazu Nezu^1^, Mami Yamazaki^1^, Hirofumi Kondo^1^, Kozo Yoneda^1^, Masaya Shimada^1^, Chigusa Yoshimura^1^, Yoko Nakano^1^, Manato Yasuda^2^, Hiroyuki Akamine^2^, Yosuke Onishi^2^, Hideo Handa^2^, Etsuko Ogaya^2^, Tomonori Kunikata^1^, Ryutaro Nakashima^1^, Hidenori Takahashi^1^, Tomohiro Nishizawa^1^, Satoshi Kuwabara^2^, Kiyosumi Takaishi^1^*

1. Shinagawa R&D Center, Daiichi Sankyo Co., Ltd., 1-2-58 Hiromachi, Shinagawa-ku, Tokyo 140-8710, Japan
2. Department of Neurology, Graduate School of Medicine, Chiba University, 1-8-1 Inohana, Chuo-ku, Chiba-shi, Chiba 260-8670, Japan

**Corresponding author:**

* Kiyosumi Takaishi

**Email:** kiyosumi.takaishi@daiichisankyo.com

**This PDF file includes:**

Supporting Information Text

Supplemental figures S1 to S9

**Supporting Information Text**

**Methods**

**Pharmacokinetics**

Pharmacokinetic studies were conducted in A/J mice obtained from Japan SLC Inc., following animal protocols approved by the Institutional Animal Care and Use Committee of Daiichi Sankyo Co., Ltd. (No. 20000135). Mice received a single subcutaneous dose of either Ab#03-H or Ab#40-H at 10 mg/kg. Blood samples were collected from the tail vein at the following time points: 1, 6, 24, 72, 168, and 360 h. The serum samples were analyzed by a Gyrolab-based sequential flow-through immunoassay method (Gyros Protein Technologies), with hMuSK Fz peptide (in-house) as the capture reagent and anti-human IgG conjugated to Alexa Fluor 647 (SouthernBiotech, #2049-31) as the detection reagent.

**Active immunization for development of MuSK-MG mouse model**

Female A/J mice were obtained from CLEA Japan, Inc. Experiments were initiated when the mice were 7 weeks of age. Mice were housed under specific pathogen-free conditions with a 12-h light/dark cycle and free access to food and water. Animal welfare was assessed by monitoring body weight, general appearance, activity, and signs of distress throughout the study. On day −13, female A/J mice were anesthetized and intradermally immunized with 6 μg of MuSK Ig1 peptide emulsified in complete Freund’s adjuvant. On day 0, immunized mice were boosted subcutaneously with 6 μg of MuSK Ig1 peptide emulsified in incomplete Freund’s adjuvant. Non-immunized mice received an equal volume of PBS in the same manner and on the same schedule. Serum anti-MuSK IgG titers were measured by ELISA using plates coated with recombinant rat MuSK. The numbers of mice were as follows: non-immunized group, n = 5; MuSK-MG groups, n = 8 per group. After confirmation of serum anti-MuSK IgG titers, immunized mice were assigned to receive Ab#03-H at 10 or 30 mg/kg by subcutaneous injection on day 1. Wire-hanging tests, EMG, and histological analyses were performed as described in the Methods.

**Purification of total IgG from patient serum**

Serum samples with a volume of 200 μL were mixed with equilibrated MabSelect PrismA (Cytiva, #17549802) and diluted fivefold in PBS (Wako, #164-23551). The mixture was incubated for 30 min at room temperature with rotation to capture IgG in serum, and was then transferred to a disposable column (Bio-Rad, #7311550). After discarding the flow-through fraction, the resins containing IgG were washed with 8 column volumes of PBS. Total IgG was eluted with 0.1 M citrate buffer (pH 3.8) and the collected fractions were immediately pH-neutralized with 1 M Tris-HCl (pH 9.0). Elution fractions corresponding to 20 column volumes of PBS were concentrated using Amicon Ultra 15 mL centrifugal devices with Ultracel 10,000 Dalton molecular weight cut-off filters (Millipore, #UFC9100). The concentration of IgG was determined using the Easy-Titer Human IgG (H+L) Assay Kit (Thermo Fisher, #23310), in accordance with the manufacturer’s instructions. SDS-PAGE was used to evaluate the quality of purified fractions.

**Immunoglobulin isotyping**

IgG levels in mouse sera, corrected at preimmunization and 9 days after booster immunization, were analyzed using the BioLegend Mouse Immunoglobulin Isotyping Panel Kit. All sera were prepared in accordance with the manufacturer’s instructions, and quantification of each isotype of mouse IgG (IgG1, IgG2a, IgG2b, IgG3, and total IgG) was performed using FlowJo software.

**Supplemental figures**

**Supplemental figure1**: (**A**) Expression of recombinant hMuSK protein in 293A cells. Full-length of hMuSK ECD domain or truncated versions of it were expressed in 293A cells and detected by immunoblotting. The table shows the quantification of immunoblot bands Schematic illustration of the full-length and truncated MuSK is shown. (**B**) The in-house antibody used in Figure 2 was added for 8 h, and AChR clustering in C2C12 cells was assessed. (**C**) C2C12 cells were treated with agrin or Ab#40-H for 4 or 24 h, and AChR clustering was assessed. All bar graphs show the mean ± SD.

**Supplemental figure 2**: Pharmacokinetics of Ab#03 and Ab#40. Following a single injection of Ab#03-H or Ab#40-H (10 mg/kg) into A/J mice, the level of antibody in the serum was evaluated.

**Supplemental figure 3**: Total IgG isotyping in active immunized mice. Concentrations of each IgG isotype were evaluated in non-immunized and immunized C57BL/6 mice at 9 days post-immunization. The bar graphs present the mean ± S.D. (n=3 in non-immunized group, n=8 in immunization group). The pie charts present the changes in the population of immunoglobulin subclasses between non-immunized and immunized mice.

**Supplemental figure 4:** (**A**) Results of the wire hanging test for each animal. Individual lines representing changes in cumulative latency over the experimental period and the dotted line indicating the mean change within each group. While most mice in the non-immunized group maintained the cumulative latency of 450 seconds, most mice in the Ig1 + IgG control group exhibited a decline (Statistical significance (p value < 0.05) was observed for the latency between the PBS control group and the Ig1 + IgG control group on days 14, 21, and 33, as determined by Welch’s t-test.). (**B**) Experimental design to analyze the effects of Ab#03-H in MuSK-MG model A/J mice. Seven-week-old A/J mice immunized with the Ig1 peptide were treated with Ab#03-H or PBS control by twice-weekly subcutaneous injections. Muscle strength and neuromuscular function were assessed using the wire hanging test and electromyography. (**C**) Serum anti-MuSK antibody levels in mice immunized with the MuSK Ig1 peptide and in naïve mice were measured by ELISA. The graph shows the mean ± SE (n = 8 for the MuSK-immunized group and n = 5 for the naïve group). (**D**) Body weight and (**E**) muscle strength, assessed by the wire hanging test, were measured over time. The graphs show the mean ± S.E. (n = 8 for each immunized group and n = 5 for non-immunized group; Shirley-Williams test comparing the Ig1 + IgG control-treated and Ig1 + Ab#03-H-treated groups; *P < 0.05). (**F**) The amplitude of the 10th compound muscle action potential (CMAP) was recorded in the hindlimb during 40-Hz nerve stimulation at the indicated time points, and the decremental response was calculated. The graphs show the mean ± SE (n = 8 for each immunized group and n = 5 for non-immunized group). (**G**) Quantification of AChR cluster intensity in isolated whole soleus muscles from each group. The bar graph shows the mean ± SE (n = 5–8 in each group; Welch’s t-test comparing the PBS control and Ig1 + IgG control-treated groups; n.s., not significant).

**Supplemental figure 5**: Generation of C2C12 myotube expressing hMuSK. (**A**) Schematic illustration of the generation of C2C12 myotube expressing hMuSK. (**B**) MuSK protein in lysates from WT C2C12, C2C12-KO, and C2C12 myotube expressing hMuSK was detected by immunoblotting. (**C**) Representative images of the AChR clustering assay in C2C12 myotube expressing hMuSK and mouse MuSK-KO C2C12 myotubes and (**D**) associated quantification are shown. AChRs were stained with fluorophore-conjugated α-BTX (green). The mCherry-positive cells represent transfected cells.

**Supplemental figure 6**: Impact of agonistic anbibody on AChR clustering recovery in the presence of MuSK autoantibodies. (**A**) The number of AChR clusters normalized to agrin-treated controls was summarized in this table. “#” indicates a statistically significant difference between agrin-treated and agrin + serum-treated groups (P < 0.05, Dunnett’s test), while “*” denotes significance between serum-treated and serum + Ab#40-H-treated groups (P < 0.05). (**B**) Representative images of C2C12 myotube expressing hMuSK treated with Ab#40-H in the presence of sera from MuSK-MG patients are shown. AChRs were stained with fluorophore-conjugated α-BTX (green). Scale bar, 100 μm.

**Supplemental figure 7**: Effect of autoantibody-free pooled serum on AChR clustering assay. Quantification of the number of AChR clusters in C2C12 myotube treated with serum from healthy individuals at the indicated concentrations. The bar graph illustrates the mean ± S.D. from three independent experiments.

**Supplemental figure 8**: Purification of IgG from MuSK-MG patient sera. (**A**) IgG from MuSK-MG patient sera was detected by SDS-PAGE. FT, flowthrough. (**B**) Yields of total IgG and (**C**) IgG4 purified from each serum sample are shown.

**Supplemental figure 9**: Evaluation of dose-dependent autoantibody binding to hMuSK in sera from patients with MuSK-MG. Serum samples from MuSK-MG patients (Patient 6 and Patient 11) were diluted from 0% to 10% and applied to 293A cells expressing full-length human MuSK (hMuSK). The binding of autoantibodies to hMuSK at each dilution was quantitatively measured using the cell ELISA.
